# Characterization of a long overlooked copper protein from methane- and ammonia-oxidizing bacteria

**DOI:** 10.1038/s41467-018-06681-5

**Published:** 2018-10-15

**Authors:** Oriana S. Fisher, Grace E. Kenney, Matthew O. Ross, Soo Y. Ro, Betelehem E. Lemma, Sharon Batelu, Paul M. Thomas, Victoria C. Sosnowski, Caroline J. DeHart, Neil L. Kelleher, Timothy L. Stemmler, Brian M. Hoffman, Amy C. Rosenzweig

**Affiliations:** 1 Departments of Molecular Biosciences and Chemistry, Northwestern University, Evanston, 60208 IL USA; 2 Department of Pharmaceutical Sciences, Wayne State University, Detroit, 48201 MI USA

## Abstract

Methane-oxidizing microbes catalyze the oxidation of the greenhouse gas methane using the copper-dependent enzyme particulate methane monooxygenase (pMMO). Isolated pMMO exhibits lower activity than whole cells, however, suggesting that additional components may be required. A pMMO homolog, ammonia monooxygenase (AMO), converts ammonia to hydroxylamine in ammonia-oxidizing bacteria (AOB) which produce another potent greenhouse gas, nitrous oxide. Here we show that PmoD, a protein encoded within many *pmo* operons that is homologous to the AmoD proteins encoded within AOB *amo* operons, forms a copper center that exhibits the features of a well-defined Cu_A_ site using a previously unobserved ligand set derived from a cupredoxin homodimer. PmoD is critical for copper-dependent growth on methane, and genetic analyses strongly support a role directly related to pMMO and AMO. These findings identify a copper-binding protein that may represent a missing link in the function of enzymes critical to the global carbon and nitrogen cycles.

## Abstract (web summary)

Methane- and ammonia-oxidizing bacteria use the integral membrane, copper-dependent enzymes particulate methane monooxygenase (pMMO) and ammonia monooxygenase (AMO) to oxidize methane and ammonia. Here the authors structurally characterize the copper-binding protein PmoD, which contains an unusual CuA site and their genetic analyses strongly support a pMMO and AMO related function of PmoD.

## Introduction

Methane-oxidizing microbes^1^ are nature’s primary sink for methane, which is the second most abundant greenhouse gas and has a global warming potential ~84 times that of CO_2_ over a 20-year period^2^. The first metabolic step carried out by methane-oxidizing bacteria (MOB), the oxidation of methane to methanol, is catalyzed by methane monooxygenase (MMO) enzymes^3^. Understanding how MMOs break a 105 kcal mol^−1^ ^4^ methane C-H bond is central to efforts to develop biological gas-to-liquid technologies^5^. The primary MMO in nature is particulate MMO (pMMO), a copper-dependent, integral membrane enzyme composed of three subunits, PmoB, PmoA, and PmoC^3,5^. A pMMO homolog, ammonia monooxygenase (AMO), converts ammonia to hydroxylamine in ammonia-oxidizing bacteria (AOB)^6^ which produce another potent greenhouse gas, nitrous oxide^7^.

**Fig. 1:**
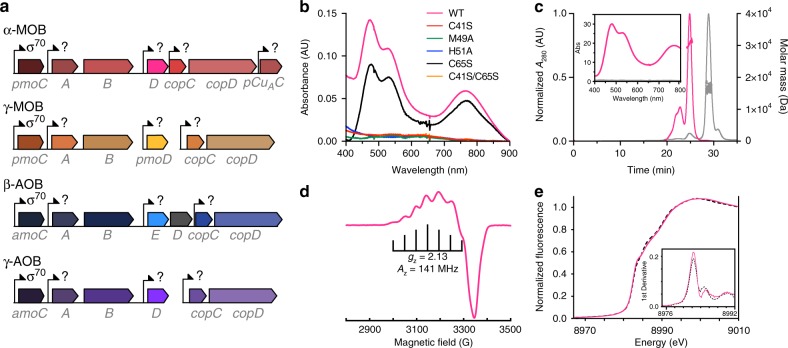
PmoD_*Met49242_1452*_ forms a dimeric species containing a Cu_A_ site. **a** Connections between *pmo*/*amo*, *pmoD*/*amoD*, *amoE*, *copCD*, and *pCu*_*A*_*C* genes in a range of AOB and MOB species. **b** Optical spectra of wild-type PmoD_*Met49242_1452*_ periplasmic domain (WT) and variants thereof (200 μM protein). All samples were loaded with ~2 equivalents copper and desalted prior to data collection. **c** SEC-MALS profiles for the two species isolated by size exclusion chromatography. Inset shows optical spectra collected at the apex of the predominant peak. Pink traces indicate the Cu_A_ species (molar mass 30,540 Da ± 2%) and gray traces indicate the monomeric species (molar mass 16,250 Da ± 3%). **d** CW X-band EPR spectra of Cu_A_ PmoD_*Met49242_1452*_; *g*_x_ = 2.01, *g*_y_ = 2.05, *g*_z_ = 2.13. Brackets define the Cu hyperfine splitting at *g*_z_ (*A*_z_). Conditions: 9.364 GHz microwave frequency, 16 G modulation amplitude, 160 ms time constant, 90 s per scan, temperature 20 K. **e** Cu XANES spectra for wild-type (solid line) and C65S (dashed line) PmoD_*Met49242_1452*_. Inset shows the first derivatives of both XANES spectra identifying features at 8983.2 and 8987.8 eV (first inflection energies at 8982.5 and 8985.3 eV) typical for mixed valent Cu(+1.5)–Cu(+1.5) species

Studies of pMMO and AMO have been hindered for the past 30 years by low enzymatic activity upon isolation. pMMO activity decreases 10- to 100-fold upon membrane isolation and protein purification^3^, and a purified AMO sample with enzymatic activity has never been obtained^8^. The persistent difficulties in obtaining high-activity pMMO/AMO preparations may be due to the absence of unidentified protein components that facilitate loading, assembly, and stabilization of the active sites and/or delivery of electrons and protons. Additional genes encoded by the *pmo* and *amo* operons provide potential candidates for such missing links. In alpha-proteobacterial MOB (α-MOB), including the *Methylosinus* and *Methylocystis* genera, and in beta-proteobacterial AOB (β-AOB), including the *Nitrosomonas, Nitrosospira*, and *Nitrosovibrio* genera^9^, the gene denoted *pmoD/amoD* is adjacent to the genes encoding the three pMMO/AMO enzyme subunits (Fig. 1a), and is partially co-regulated with *pmoCAB* and *amoCAB*^10–12^. In beta-proteobacterial *amo* operons, an additional gene homologous to *amoD*, denoted *amoE*/*orf4*, precedes *amoD* (Fig. 1a)^9^. These extended operons also encode CopC, CopD, and sometimes PCu_A_C proteins, all of which have been implicated in copper homeostasis in a range of bacteria^10^. Gamma-proteobacterial *pmo*/*amo* operons contain fewer genes: in *Nitrosococcus* genera, only *amoD* follows *amoCAB*, and in related gamma-proteobacterial methanotrophs, including the *Methylococcus* genera, *pmoD* genes are present, but are not found in *pmo* operons^11,13^. Most MOB and AOB genomes contain at least two *pmoD/amoD* homologs, including those of MOB from the verrucomicrobial and NC-10 subgroups and AOB belonging to the *Nitrospirae* phylum, although not archaeal ammonia oxidizers. Here we show that PmoD forms a copper center exhibiting the features of a Cu_A_ site using a previously unobserved ligand set at the interface of a PmoD homodimer. We further show that PmoD is critical for the copper-dependent growth of *Methylosinus trichosporium* OB3b on methane. Finally, genetic analyses support a role for PmoD/AmoD related to the function of pMMO/AMO.

## Results

### PmoD forms a Cu_A_ site as a homodimer

Sequence analysis indicates that PmoD/AmoD comprises an N-terminal signal peptide followed by a periplasmic domain containing two strictly conserved cysteine residues and a C-terminal transmembrane helix within the inner membrane (Supplementary Figure 1a). We heterologously expressed and purified the periplasmic domain of PmoD encoded by the *Methylocystis* species (sp.) strain Rockwell extended *pmo* operon (PmoD_*Met49242_1452*_; there are 10 other *pmoD* genes in the genome of this species) (Supplementary Figure 1b, d). Addition of CuSO_4_ to PmoD_*Met49242_1452*_ after reduction with dithiothreitol (DTT) gives rise to a species characterized by an intense purplish pink color. The absorption spectrum of this species exhibits peaks at 475, 530, and 770 nm, resembling spectra of the Cu_A_-containing domains of cytochrome *c* oxidase (C*c*O), nitrous oxide reductase (N_2_OR), and engineered Cu_A_ proteins, including Cu_A_ azurin^14^ (Fig. 1b). The copper-loaded protein can be further resolved into two distinct species by size exclusion chromatography (Supplementary Figure 1c). The first of these retains the Cu_A_-like spectral features and contains 1.4 ± 0.2 equivalents of Cu per protein molecule, while the second lacks the Cu_A_-like spectral features and contains 3.1 ± 1.2 equivalents of Cu per protein molecule. Each was further analyzed using size exclusion chromatography with multi-angle light scattering (SEC-MALS): the Cu_A_-like species has a molar mass of 30,540 Da (±2%), consistent with a PmoD_*Met49242_1452*_ dimer, while the second species has a molar mass of 16,250 Da (±3%), consistent with a PmoD_*Met49242_1452*_ monomer (Fig. 1c).

Cu_A_ centers feature a mixed valent Cu(+1.5)–Cu(+1.5) site in which two copper ions separated by ~2.5 Å are coordinated by two bridging cysteine thiolates, two in-plane histidine imidazoles, and a methionine thioether and backbone carbonyl as axial ligands. The canonical Cu_A_ amino acid ligands derive from a conserved Hx_34_Cx_3_Cx_3_Hx_2_M motif^15^ which is not present in PmoD sequences. In typical Cu_A_ centers, the 475 nm and 530 nm absorption maxima are assigned to sulfur (Cys) to Cu ligand-to-metal charge transfer transitions, and the longer wavelength near-infrared feature (~770 nm) arises from the Ψ → Ψ* Cu–Cu bonding to antibonding orbital transition^16^. The energy of this transition is inversely correlated to the length of the Cu–Cu bond^14^. In the PmoD_*Met49242_1452*_ spectrum, this feature is blue-shifted relative to natural Cu_A_ systems (775–808 nm for C*c*O and 780–800 nm and N_2_OR)^14^ and is more similar to that observed for the semisynthetic Cu_A_ azurin (765 nm)^17^, indicating that PmoD_*Met49242_1452*_ has a short Cu–Cu distance with strong Cu–Cu bonding. The presence of a Cu_A_ site with a Cu_2_S_2_ core is definitively established by the PmoD_*Met49242_1452*_ electron paramagnetic resonance (EPR) spectrum which exhibits the characteristic seven hyperfine line splitting along *g*_*z*_ (Fig. 1d)^18^, defining a fully valence-delocalized dicopper center. However, the *A*_z_ and *g*_z_ values for the PmoD_*Met49242_1452*_ Cu_A_ site (*A*_z_ = 141 MHz, *g*_z_ = 2.13) are significantly smaller than those of Cu_A_ azurin (*A*_z_ = 167 MHz, *g*_z_ = 2.17^19^), while compared to C*c*O and N_2_OR (*A*_z_ = 107-117 MHz, *g*_z_ = 2.18, *A*_z_ = 117 MHz, *g*_z_ = 2.18, respectively)^14^, the *A*_z_ is larger and the *g*_z_ is smaller. The PmoD *g*_z_ = 2.13 is the smallest observed for a Cu_A_ site to date^14^. By contrast, the monomeric species of PmoD_*Met49242_1452*_ exhibits an optical spectrum with no charge transfer bands (Fig. 1c, inset) and is EPR silent. The X-ray absorption near edge structure (XANES) spectra of PmoD_*Met49242_1452*_ exhibit features similar to those observed for Cu_A_ model compounds and the soluble Cu_A_ domain of *Thermus* *thermophilus* C*c*O^20^ (Fig. 1e, Supplementary Discussion). In addition, extended X-ray absorption fine structure (EXAFS) data can be fit with a 2.41 Å Cu–Cu interaction (Supplementary Discussion, Supplementary Figure 2a-d, Supplementary Table 1), consistent with the observed absorption spectrum.

### PmoD uses a non-canonical ligand set to form the Cu_A_ site

**Fig. 2:**
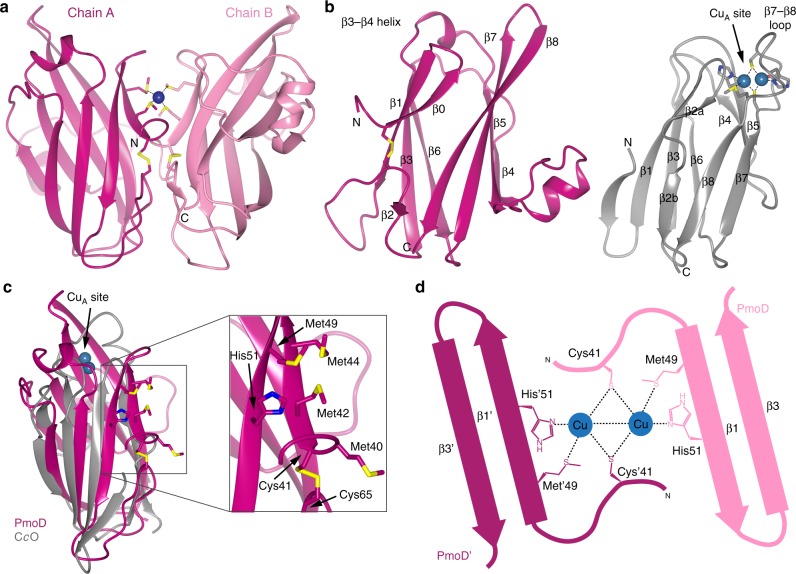
Structural comparison of PmoD_*Met49242_1452*_ to Cu_A_-containing domains. **a** Crystal structure of PmoD_*Met49242_1452*_ periplasmic domain. The asymmetric unit of the crystal is shown as a ribbon diagram, with chain A in magenta and chain B in pink. The copper ion is depicted as a blue sphere and the coordinating residues as well as the two invariant cysteines are shown in stick format. **b** Comparison of PmoD_*Met49242_1452*_ structure (left; magenta) to the Cu_A_-containing domain of *T. thermophilus* C*c*O (PDB: 2CUA). Secondary structure elements are labeled, and the Cu_A_ site in C*c*O is indicated with Cu ions shown as spheres and coordinating ligands in stick format. **c** Superposition of PmoD_*Met49242_1452*_ periplasmic domain (magenta) with C*c*O (gray) colored as in (**b**). The box indicates the region containing the PmoD_*Met49242_1452*_ candidate metal binding ligands, and the inset shows a close-up of the same, shown in stick format. **d** Model depicting how PmoD_*Met49242_1452*_ can form a Cu_A_-like site as a dimer. The two molecules are shown as pink and magenta cartoons, with the residues identified as ligands indicated

Both the dimeric and monomeric species of PmoD_*Met49242_1452*_ were subjected to crystallization trials, but crystals were obtained from only the monomeric form. The 1.9 Å resolution structure solved by copper single wavelength anomalous dispersion (SAD) phasing (Supplementary Table 2, Supplementary Figure 3a) reveals a cupredoxin fold^14^ with a single copper ion located between the two PmoD_*Met49242_1452*_ monomers in the asymmetric unit. The copper ion is coordinated in tetrahedral geometry by Met42 and Met44 from each monomer, and the two invariant cysteine residues, Cys41 and Cys65, form an intramolecular disulfide bond (Fig. 2a and Supplementary Figure 3b, c). Given that the crystals were obtained from the monomeric PmoD_*Met49242_1452*_ species, it is likely that the observed copper-binding site is a crystallization artifact. Nevertheless, comparison of the overall structure to other Cu_A_-containing domains shows that the Cu_A_ site in PmoD_*Met49242_1452*_ must be formed in a unique fashion.

The coordinates of PmoD_*Met49242_1452*_ can be superposed on those of the Cu_A_ domains of C*c*O^21^ (2CUA (10.2210/pdb2cua/pdb]) and N_2_OR^22^ (1FWX (10.2210/pdb1fwx/pdb)), as well as the engineered azurin Cu_A_ domain^23^ (1CC3 (10.2210/pdb1cc3/pdb)) with root-mean-square deviation (r.m.s.d.) values of 3.06, 2.82, and 2.95 Å for 82, 80, and 82 Cα atoms, respectively (Supplementary Figure 3d, e). The PmoD_*Met49242_1452*_ structure differs from these canonical Cu_A_ domain structures in three distinct regions (Fig. 2b and Supplementary Figure 3b, c). First, the N-terminal histidine ligand in the Hx_34_Cx_3_Cx_3_Hx_2_M Cu_A_ motif typically derives from the loop between β3 and β4, but PmoD_*Met49242_1452*_ contains a small helix in place of this loop and lacks a histidine in the equivalent position. Second, the loop between β7 and β8 provides the other four Cu_A_ ligands, but PmoD_*Met49242_1452*_ lacks not only this sequence motif, but also any histidine, cysteine, or methionine residues in this area. Instead, this region in PmoD_*Met49242_1452*_ is an extended β hairpin (Fig. 2b). Finally, the N-terminal region of PmoD_*Met49242_1452*_ diverges in the orientation of β1 and β2, as well as the presence of an additional β strand prior to β1. These changes at the N terminus are likely due to the intramolecular disulfide bond (Supplementary Figure 3b).

Instead of the canonical Cu_A_ ligands, PmoD_*Met49242_1452*_ contains a cluster of surface-exposed cysteine, methionine, and histidine residues near its N terminus, including the universally conserved cysteine residues Cys41 and Cys65 (Fig. 2c). A variant of PmoD_*Met49242_1452*_ in which Cys65 is replaced with serine retains the Cu_A_ features (Fig. 1b,e, Supplementary Figure 2e-h), and exhibits nearly complete dimerization upon addition of copper (Supplementary Figure 1c) with 0.80 ± 0.02 equivalents of Cu bound per protein molecule. While these data indicate that Cys65 is not a Cu_A_ ligand, the reduced copper stoichiometry compared to wild type indicates that Cys65 may bind an additional copper ion. By contrast, replacement of Cys41 with serine abolished appearance of the Cu_A_ spectral features. Since PmoD_*Met49242_1452*_ only contains these two conserved cysteine residues and Cu_A_ sites require two bridging thiolates, the Cu_A_ site must be formed between two PmoD_*Met49242_1452*_ monomers, of which each contributes a Cys41 ligand. The periplasmic domain of PmoD_*Met49242_1452*_ contains four histidine residues, of which one, His51, is located near Cys41 (Fig. 2c). Replacement of His51 with alanine also eliminates Cu_A_ formation, as does replacement of Met49 with alanine (Fig. 1b), implicating these two residues as ligands. Thus, despite the overall similarity in fold to other Cu_A_ domains, the PmoD Cu_A_ site is formed by two protein molecules and a completely different part of the protein structure (Fig. 2d) in contrast to all previously characterized Cu_A_ centers which are housed within a single cupredoxin domain.

### Disruption of *pmoD* in vivo results in a copper-dependent growth defect

**Fig. 3:**
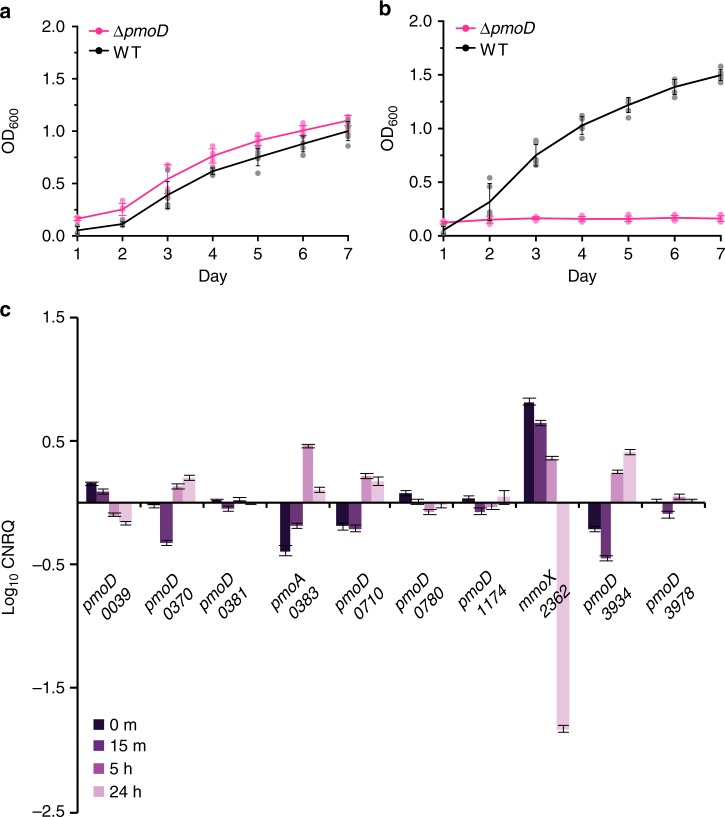
In vivo characterization of PmoD_*MettrDRAFT_0381*_. **a** Growth curves of wild-type (black) and Δ*pmoD* (pink) *Methylosinus trichosporium* OB3b under copper starved conditions. Error bars represent s.d., *n* = 5–6. **b** Growth curves of wild-type (black) and Δ*pmoD* (pink) *Methylosinus trichosporium* OB3b grown in the presence of 10 μM CuSO_4_. Error bars represent s.d., *n* = 5–6. **c** qPCR analysis of *pmoD* genes in *Methylosinus trichosporium* OB3b at four timepoints (0 min at 0 μM CuSO_4_, 15 min at 12.5 μM CuSO_4_, 5 h at 12.5 μM CuSO_4_, 24 h at 12.5 μM CuSO_4_)_._ Regulatory changes are shown as log_10_-transformed normalized relative quantities (NRQs). The four-digit code beneath the gene name represents the locus tag (in MettrDRAFT_NNNN format). Two non-*pmoD* genes are included for comparison: components of the two MMOs, pMMO (*pmoA_0383*) and sMMO (*mmoX_2362*), which are reciprocally regulated by copper. The clearest copper-responsive regulation is observed for the *copC*-adjacent *pmoD* MettrDRAFT_0039 (the genes are both copper down-regulated, and regulatory changes are correlated with those observed for components of the *mmo* and *mbn* operons) and for the *pCu*_*A*_*C*-adjacent *pmoD* MettrDRAFT_3934 (the genes are both copper up-regulated, and regulatory changes are correlated with those observed for components of the *pmo* operon and *csp1*). Error bars represent standard error between three biological replicates, each containing three technical replicates. Data points are shown in Supplementary Figure 5

To assess the role of PmoD in vivo, we disrupted the *pmoD* gene in the extended copy of the *pmo* operon (containing *pmoCAB* as well as the *pmoD*, *copC*, *copD*, and *pCu*_*A*_*C* genes) in *Methylosinus trichosporium* OB3b (PmoD_*MettrDRAFT_0381*_). Unlike *Methylocystis* sp. Rockwell, *Methylosinus trichosporium* OB3b also possesses genes for the soluble, iron-containing MMO (sMMO) and for proteins related to the biosynthesis, regulation, and transport of the siderophore-like copper chelator methanobactin (Mbn)^24^. Under copper starvation conditions, sMMO and Mbn are produced; both processes are associated with characteristic phenotypes. When copper is bioavailable, these genes are repressed as part of the so-called copper switch, and the pMMO genes are mildly up-regulated^10^. Growth of both the wild-type and Δ*pmoD* strains was monitored in either copper-free media or media supplemented with 10 μM CuSO_4_. Under copper starvation conditions, Δ*pmoD Methylosinus trichosporium* OB3b is phenotypically similar to the wild-type strain. The strains grow at a comparable rate (Fig. 3a), and both produce Mbn (Supplementary Figure 4c, d), exhibit sMMO activity (Supplementary Figure 4e), and produce sMMO (Supplementary Figure 4f). Under copper-replete conditions, however, Δ*pmoD Methylosinus trichosporium* OB3b exhibits a pronounced growth defect. Cultures grown in copper-supplemented media produce significantly less cell density after 1 week of growth (Fig. 3b), and the Δ*pmoD* strain also exhibits a growth defect on nitrate mineral salts (NMS) agar plates supplemented with CuSO_4_ (Supplementary Figure 4a, b). This phenotype is distinct from the wild-type strain which exhibits a slight increase in growth rate under copper-replete conditions^25^ (Fig. 3b). Despite the growth defect in the presence of copper, the Δ*pmoD* strain, like the wild-type strain, does not produce Mbn (Supplementary Figure 4g, h), and no longer exhibits sMMO activity (Supplementary Figure 4i). While the sMMO subunits are significantly less abundant in copper-replete cell lysates, the pMMO subunits are detected in both the wild-type and Δ*pmoD Methylosinus trichosporium* OB3b strains (Supplementary Figure 4j). Thus, PmoD_*MettrDRAFT_0381*_ does not appear to play a role under sMMO-utilizing conditions, but is important for growth under pMMO-utilizing conditions. It is not clear, however, whether the Cu_A_ site is linked to the observed growth defect.

### PmoD homologs exhibit varying levels of copper-dependent regulation

Interestingly, this copper-dependent growth defect is observed despite the presence of seven additional PmoD homologs encoded in the *Methylosinus trichosporium* OB3b genome, suggesting that all PmoD proteins are not functionally equivalent. We previously showed that the components of the full *Methylosinus trichosporium* OB3b *pmo* operon (*pmoCAB, pmoD*, *copC*, *copD*, and *pCu*_*A*_*C*) are very mildly up-regulated by copper^10^, and other studies have shown that in AOB, *amoD* (and, if present, *amoE*) are co-up-regulated and co-transcribed with *amoCAB* upon exposure to ammonia^12,26^. The regulation of *pmoD* homologs beyond the *pmo* operon has not been investigated. Therefore, we conducted time-dependent, copper-responsive quantitative polymerase chain reaction (qPCR) analysis of all *Methylosinus trichosporium* OB3b non-operon *pmoD* genes. We observe varying patterns of copper-dependent regulation, with some *pmoD* genes mildly repressed, some up-regulated, and several not regulated by copper at all (Fig. 3c and Supplementary Figure 5), further supporting that not all PmoD proteins are functionally equivalent. A recent RNA-sequencing study on *Methylosinus trichosporium* OB3b that lacks the time-resolved component of these experiments is nevertheless consistent with the observed regulatory patterns for *pmoDs* with significant regulatory changes and/or correlated co-regulation with other copper-responsive genes^27^. Moreover, differential regulation of *pmoD* genes in response to copper is also observed for *Methylococcus capsulatus* (Bath)^28^ which has two *pmoD* genes of which neither is located in its *pmo* operon. These regulatory patterns combined with the Δ*pmoD Methylosinus trichosporium* OB3b phenotype suggest that PmoD homologs play distinct roles in vivo.

In support of nonredundant functionality, the production of many PmoD homologs is evident by proteomics at variable levels. We detected PmoD soluble domains and predicted C-terminal helices, but not the N-terminal signal peptides, in membrane fractions or whole cell lysate from five different methanotrophs (Supplementary Figure 6). For *Methylocystis* sp. Rockwell, 5 of the 11 PmoD homologs are observed in solubilized membrane fractions, including PmoD_*Met49242_1452*_ (Supplementary Figure 6a). In solubilized membrane fractions from *Methylomicrobium buryatense* 5GB1C (Supplementary Figure 6b) and *Methylomicrobium alcaliphilum* 20Z (Supplementary Figure 6c), whose genomes each contain two PmoD homologs, both homologs are detected. In the case of *Methylococcus capsulatus* (Bath) (Supplementary Figure 6d), which also has two PmoD homologs, one was observed. In whole cell lysate from *Methylosinus trichosporium* OB3b grown under copper-replete conditions, six of eight PmoD homologs are observed, including those encoded by genes adjacent to *copCD*, *pCu*_*A*_*C*, and *pmoCAB* (Supplementary Figure 6e).

### Bioinformatics analyses suggest functional differences

**Fig. 4:**
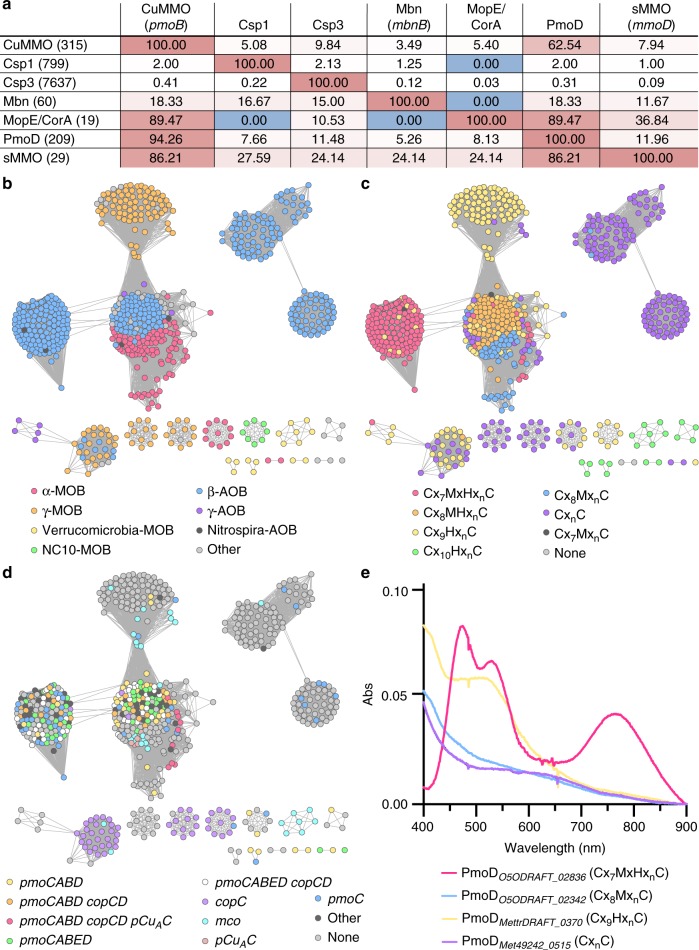
PmoD subfamilies and their connections to copper homeostasis. **a** Genomes present in the JGI/IMG database were analyzed for the presence of genes with a proposed relationship to the methanotroph copper homeostasis, including *csp1*/*2* (TIGR04401), copper membrane monooxygenases (CuMMOs) via *a*/*pmoB* (PF04744/TIGR03079), Mbn via *mbnB* (TIGR04159), *pmoD* (the dataset in this paper), *csp3*/DUF326 (PF03860), *mopE*/*corA* (via BLAST, no profile HMM available), and sMMO via *mmoD* (TIGR04550, absent in non-sMMO soluble diiron monooxygenase enzymes). The strongest reciprocal relationship is observed between PmoDs and pMMO components. **b** A sequence similarity network (SSN) for PmoD homologs was constructed using the EFI/EST network generation tool and an *E*-value cutoff of 21, colored by AOB/MOB distribution among PmoD homologs. **c** SSN colored by core cysteine-containing sequence motifs among PmoD homologs. The Cx_7_MxHx_n_C motif (pink) corresponds to the PmoD_*Met49242_1452*_ Cu_A_ site ligand set and is also present in AmoEs. **d** SSN colored by proximity to copper-related genes, including *amoCAB*/*pmoCAB* operons (*pmo* operons), lone *amoC*/*pmoC* genes (*pmoC*), *copCD* pairs or lone *copC* genes, *pCu*_*A*_*C* genes, genes encoding multicopper oxidases, and genes encoding additional *pmoD* homologs. **e** Optical spectra of representative classes of copper-loaded PmoD homologs denoted by locus tag and metal binding motif

To address potential functional differences among PmoD homologs, we performed a detailed bioinformatic analysis of all PmoD sequences identifiable in the Joint Genome Institute/Integrated Microbial Genomes (JGI/IMG) genome database. The vast majority of these are found in MOB and AOB, and all are found in species with genomes containing at least one operon encoding a copper membrane monooxygenase (CuMMO) related to pMMO/AMO (Fig. 4a). Notably, the only genomes that encode pMMO/AMO homologs, but not PmoD proteins, are species that oxidize neither methane nor ammonia such as strains of *Mycobacteria* and *Nocardiodes*^29^ (Fig. 4b). The number of PmoD homologs per genome ranges from one (e.g., *Methylovulum* and *Methylogaea* species, and some species outside of canonically methanotrophic genera) to 11 (*Methylocystis* sp. Rockwell), all of which differ in sequence and genomic context. Most canonical MOB and AOB genomes contain at least two PmoD homologs. An alignment of all putative PmoD amino acid sequences was used to construct a preliminary PmoD profile hidden Markov model (HMM) and to identify several core sequence motifs involving the two invariant cysteines and other putative metal binding residues (Supplementary Figure 7). Genomic neighborhood analysis provides additional insight into the link between the various PmoD proteins and pMMO/AMO (Supplementary Figure 8). PmoD proteins containing either a Cx_7_MxHx_n_C motif (like PmoD_*Met49242_1452*_) or a Cx_8_MHx_n_C motif comprise 23.6% and 13.4%, respectively, of all PmoDs (Fig. 4c, Supplementary Figure 7). These subsets are predominantly associated with *pmo*/*amo* operons (the latter annotated in AOB as *amoD* and the former as *amoE*) and are also found in the proximity of some lone *pmoC* genes (Fig. 4d). In α-MOB, the *pmoD* genes found in *pmo* operons encode PmoDs with the Cx_7_MxHx_n_C motif, as observed in the proteins encoded by *amoE* and not in *amoD*. The close association between genes encoding PmoD and pMMO/AMO is markedly different from the neighboring genes encoding CopCs, CopDs, and PCu_A_Cs which are quite common in other prokaryotes^30^. These in-operon PmoDs are likely to have functions specific to pMMO and AMO, consistent with the phenotype observed in the Δ*pmoD Methylosinus trichosporium* OB3b strain (Fig. 3a).

### Cu_A_ site formation is limited to homologs with a Cx_7_MxHx_n_C motif

Since the ligand set identified for the Cu_A_ site in PmoD_*Met49242_1452*_ (Cys41, Met49, and His51), along with Cys65, which is not involved in the Cu_A_ site, corresponds to the Cx_7_MxHx_n_C motif (Fig. 4c, Supplementary Figure 7), we investigated whether other PmoD homologs containing this motif are also capable of forming a Cu_A_ center. We expressed and purified PmoD_*O5ODRAFT_02836*_, a homolog from *Methylocystis parvus* OBBP. According to metal quantitation and EPR and optical spectroscopic characterization, PmoD_*O5ODRAFT_02836*_, like PmoD_*Met49242_1452*_, binds copper (Supplementary Table 3) and forms a Cu_A_ species (Fig. 4e, Supplementary Table 4, Supplementary Figure 9), although a significant fraction (estimated at ~50%) of the PmoD_*Met49242_1452*_ EPR signal is attributable to mononuclear type 2 Cu(II) resonance^31,32^.

We also examined the genomic neighborhoods of genes encoding PmoDs with other putative metal binding motifs and conducted in vitro metal binding and spectroscopic experiments on representative members of these classes. The representative Cx_8_Mx_n_C motif-containing (7.9% of all PmoD homologs, commonly near genes encoding multicopper oxidases, PCu_A_Cs, and ABC transporters) and Cx_9_Hx_n_C motif-containing (22.2%, genes often found near *copC*) PmoDs *Methylocystis parvus* OBBP PmoD_*O5ODRAFT_02342*_ and *Methylosinus trichosporium*OB3b PmoD_*MettrDRAFT_0370*_ bind copper (Supplementary Table 3), but do not exhibit optical or EPR spectroscopic features consistent with a Cu_A_ site (Fig. 4e, Supplementary Figure 9). In addition, a large number of PmoDs have no conserved metal binding residues beyond the Cx_n_C motif (29.0%) and typically neighbor genes of unknown relevance or are adjacent to *copC* genes (Supplementary Figure 8). While one such PmoD, PmoD_*Met49242_0515*_, also binds copper, it too cannot form a Cu_A_ species (Supplementary Table 3, Fig. 4e, Supplementary Figure 9). Thus, Cu_A_ site formation is confined to Cx_7_MxHx_n_C-containing PmoDs encoded by genes within extended *pmo/amo* operons.

## Discussion

PmoD/AmoD represent a family of copper-binding proteins specific to MOB and AOB, the key players in global carbon and nitrogen cycling. The subset of PmoD homologs encoded in genomic proximity to and co-regulated with pMMO/AMO contains an unprecedented type of Cu_A_ site and is important for copper-dependent growth on methane. PmoD homologs are more closely correlated with CuMMOs than any other recently proposed members of the MOB/AOB copper homeostasis system, including the periplasmic copper sponge protein Csp1/2, the cytoplasmic copper sponge protein Csp3, the secreted copper-binding proteins CorA/MopE, and the copper-binding chalkophore Mbn (Fig. 4a). This observation strongly supports a unique role for PmoDs in the function of CuMMOs.

Potential functions of PmoD include providing reducing equivalents to drive pMMO reactivity, facilitating copper loading of pMMO, or even structurally stabilizing pMMO within the membrane. The specific biological role of PmoD, including whether its function in vivo is mediated via the homodimeric Cu_A_ species, remains to be determined. More comprehensive characterization of the unusual Cu_A_ site, combined with additional in vivo and in vitro studies of PmoD including its transmembrane helix, are needed to address these possibilities. Furthermore, functional differences among the entire PmoD/AmoD family remain to be elucidated. Given the prevalent genomic proximity of *pmoD*/*amoD* genes to those encoding a range of copper-containing proteins, it is likely that additional copper centers and copper-related functions will be uncovered.

## Methods

### Cloning and construct design for PmoD expression plasmids

The expression constructs for the N-terminally His_6_-tagged tobacco etch virus (TEV)-cleavable PmoD periplasmic domains were all prepared in the pSCG-His vector by Anthony Gizzi and Steven Almo (Albert Einstein College of Medicine) using the primers listed in Supplementary Table 5. The cysteine variants in PmoD_*Met49242_1452*_ were generated via a QuikChange Lightning kit as directed (Agilent), using primers listed in Supplementary Table 5. The Met49Ala and His51Ala variants were synthesized by Genscript along with a TEV cleavage site before the N-terminal residue and subcloned into the pET28a vector. All constructs include an additional vector-derived serine and methionine at the N terminus.

### Expression and purification of the PmoD periplasmic domains

All PmoD periplasmic domain constructs were expressed and purified following the same protocol. Plasmid DNA was transformed into BL21*(DE3) cells (Novagen). Overnight cultures in LB media were grown at 37 °C with agitation at 200 rpm, followed by inoculation into 1 L autoinduction media^33^ and incubation at 37 °C with agitation at 200 rpm until reaching an optical density at 600 nm of 0.6. The temperature was then reduced to 22 °C, and the cells were harvested after ~18 h by centrifugation at 6000 × *g* for 15 min. The resulting cell pellet was resuspended in lysis buffer (20 mM imidazole, 20 mM Tris, pH 8.0, 500 mM NaCl) supplemented with 1 mM DTT, 1 mg mL^−1^ DNase I (Sigma), and 1 mM phenylmethylsulfonyl fluoride dissolved in ethanol (EtOH). Cells were lysed via sonication for 15 min with 1 s pulses. Cell debris was removed by centrifugation at 24,000 × *g* for 1 h.

The resulting supernatant was then applied to NiNTA resin (Qiagen) pre-equilibrated with chilled lysis buffer. The flow-through was discarded, and the resin was washed with 5 column volumes of lysis buffer. His_6_-TEV-PmoD was eluted with 2 column volumes of elution buffer consisting of lysis buffer with 500 mM imidazole. To remove the His_6_ tag, TEV protease was added to the eluate, and the sample was dialyzed in 10 kDa molecular weight cutoff (MWCO) SnakeSkin dialysis tubing (Thermo) against 20 mM Tris, pH 7.0, and 1 mM DTT overnight at 4 °C. Following cleavage, the dialyzed sample was then re-applied to NiNTA resin to remove the TEV protease and the cleaved tag. The flow-through containing purified PmoD was then concentrated in 10 kDa MWCO Amicon spin concentrators (Millipore) and either used immediately or flash frozen in liquid nitrogen and stored at −80 °C.

### Metallation of PmoD

Purified PmoD samples were buffer exchanged by dilution into reductant-free buffer (20 mM Tris, pH 7.0) and concentrating in 10 kDa MWCO spin concentrators (Millipore) to 200–600 μM followed by the slow addition of 2 molar equivalents of CuSO_4_ with constant mixing on ice. Excess copper was then removed either using a disposable PD-10 desalting column (GE Healthcare Life Sciences) or by size exclusion chromatography using a HiLoad 16/600 Superdex 75 column (GE Healthcare Life Sciences). By the latter method, two distinct PmoD_*Met49242_1452*_ species can be resolved, the dimeric species that forms the Cu_A_-like site and a monomeric copper-bound species. All protein concentrations were measured using the Bradford assay with a standard curve generated from known concentrations of bovine serum albumin. Optical spectra were collected using copper-loaded samples containing 200 μM protein in a quartz cuvette (Helma) at room temperature using an Agilent 8453 spectrophotometer. For metal analysis, all samples were first desalted on a PD-10 column into metal-free buffer (20 mM Tris, pH 7.5) and digested in 5% nitric acid in metal-free tubes (VWR). Standard curves were generated using a dilution series of a custom multi-element standard (Inorganic Ventures). The copper content of the samples was determined using a Thermo iCAP 7600 ICP-OES instrument in the Quantitative Bio-element Imaging Center (QBIC) core facility at Northwestern University.

### Size exclusion chromatography with multi-angle light scattering

SEC-MALS was used to determine the molar masses of the two PmoD species resolved by SEC. Each peak was pooled and concentrated to 2–4 mg mL^−1^ in 10 kDa MWCO concentrators (Millipore). Samples were analyzed using an Agilent 1260 series high-performance liquid chromatography (HPLC) system equipped with diode-array detection absorbance in-line with a DAWN HELEOS II multi-angle static light scattering detector (Wyatt Technology), a QELS dynamic light scattering detector (Wyatt Technology), and a T-rEx differential refractive index detector (Wyatt Technology). Then, 300 μL protein was applied to a Superdex 75 Increase 10/300 GL column (GE Healthcare) that had been pre-equilibrated in a mobile phase consisting of 20 mM Tris, pH 7.0. The mobile phase and protein sample prior to injection were maintained at 4 °C and the column was kept at 8 °C. Each sample was run at 0.4 mL min^−1^ for 60 min. Optical spectra were also recorded at the apex of each peak. Data were processed and analyzed using Astra software version 5.3.4 (Wyatt Technology).

### Electron paramagnetic resonance spectroscopy

EPR samples were prepared in 20 mM Tris, pH 7.0, at concentrations of 100–300 μM protein. Protein solution was loaded into Wilmad quartz X-band EPR tubes (Sigma Aldrich) and samples were then frozen in liquid nitrogen, where they were stored until analysis. Continuous wave (CW) X-band spectra were collected on a Bruker ESP-300 spectrometer with an Oxford Instruments ESR-900 helium flow cryostat.

### X-ray absorption spectroscopy

The Cu_A_-like dimeric species of wild-type PmoD_*Met49242_1452*_ isolated by size exclusion chromatography was concentrated to 1.8 mM Cu (1.26 mM protein) in 20 mM Tris, pH 7.0, 30% glycerol. The C65S sample was concentrated to 900 μM Cu (740 μM protein). Each sample was loaded into a 2 mm Lucite EXAFS cell wrapped in Kapton tape, flash frozen in liquid nitrogen, and stored in liquid nitrogen until exposure to X-ray radiation. X-ray absorption spectroscopy (XAS) data were collected at the Stanford Synchrotron Radiation Lightsource on beamline 9−3 which is equipped with a Si[220] double-crystal monochromator with an upstream mirror for focusing and harmonic rejection. Fluorescence spectra were collected using a 100-element Ge solid-state detector (Canberra). During data collection, the continuous-flow liquid helium cryostat (Oxford Instruments) was stabilized at 10 K. Copper excitation data were collected using a 6 μm nickel filter and solar slits placed between the cryostat and detector to reduce scattering fluorescence. XAS spectra were measured using 5 eV steps in the pre-edge region (8750–8960 eV), 0.25 eV steps in the edge region (8986-9050 eV), and 0.05 Å^−1^ increments in the EXAFS region (to *k* = 13.3 Å^−1^, integrating from 1 to 25 s in a *k*^3^ weighted manner for a total scan length of approximately 40 min). A Cu foil spectrum was collected simultaneously with each protein spectrum for energy calibration, with an assigned first inflection point at 8980.3 eV. Spectra were closely monitored for any photodamage and slight photoreduction was observed in the second scan at each exposure position. To quantitate the extent and impact of photoreduction, individual spectra were collected at unique positions on the sample surface using a matrix positioning grid (ensuring a new face for radiation exposure), and only the initial exposure spectrum at each position was used during overall data analysis. Data represent the average of 6 scans.

XAS spectra were processed and analyzed using the EXAFSPAK program suite written for Macintosh OSX (EXAFSPAK; http://www-ssrl.slac.stanford.edu/~george/exafspak/exafs.htm, 2001), integrated with the Feff v8 software^34^ for theoretical model generation. EXAFS fitting analysis was performed on raw/unfiltered data. Single scattering models were calculated for oxygen, nitrogen, sulfur, copper, and carbon coordination to simulate possible copper ligand environments, with values for the scale factors (Sc) and E_0_ calibrated by previous fittings of characterized crystallographic copper model compounds^35^. Standard criteria for judging the best-fit EXAFS simulations included reasonable Debye–Waller factors (σ^2^ < 0.006 Å^2^)^36^, the spectral resolution calculated based on the energy range extent of usable data^37^, and the lowest mean square deviation between data and fit corrected for the number of degrees of freedom (*F’*)^37^. During the standard criteria simulations, only the bond length and Debye–Waller factor were allowed to vary for each ligand environment. A scale factor of 0.95 and E_0_ values of −12, −14, and –16 were used for Cu–C/N/O, Cu–S, and Cu–Cu theoretical model calibration; these values were obtained from simulating authentic Cu–Cu models^35^. Simulations applying a formal but static Cu–Cu interaction were also tested in an attempt to deconvolute potential Cu–Cu scattering in the Cu EXAFS for each sample set. Fits using the static Cu–Cu ligand environment followed the strategy outlined by Blackburn et al.^20^ which was applied to systems in which Cu–S and Cu–Cu interactions were nonresolvable, as is the case in these data. Static Cu–Cu fits held the Cu ligand parameters constant, manually stepping through the Cu–Cu bond length, but not allowing the bond length or Debye–Waller factor for this ligand environment to vary; however, the bond length and Debye–Waller factor in each additional ligand environment was allowed to vary during these static Cu–Cu simulations (Supplementary Table 1).

### Crystallization and structure determination

Crystals of the copper-bound monomeric species were obtained using 3 mg mL^−1^ protein in the Classics Suite (Qiagen) condition H1 (0.1 M ammonium acetate, pH 4.6, 0.2 M ammonium sulfate, 25 % (w/v) polyethylene glycol (PEG) 4000). Crystallization conditions were further optimized, and the crystal used for data collection was obtained from sitting drop screens with 0.1 M sodium acetate, pH 4.6, 0.2 M ammonium sulfate, 18% PEG 4000 as the precipitant. Prior to data collection, the crystal was transferred to a cryoprotectant solution consisting of the precipitant supplemented with 10% ethylene glycol for 5 s and then submerged in liquid nitrogen. Data were collected at 100 K at a wavelength of 1.377 Å using a Pilatus detector at GM/CA beamline 23 ID-B at the Advanced Photon Source (APS) at Argonne National Laboratory. The crystals belong to space group *P*3_2_21 with two PmoD monomers in the asymmetric unit. The data were processed using HKL2000 to 1.9 Å resolution (Supplementary Table 2). The structure was solved using the SAD method with phenix.autosol^38^, yielding a Bayes CC of 56.6 and a FOM (figure of merit) of 45.6, indicating that the solution was likely correct. Phenix.autobuild^38^ was used to obtain an initial model that included 245 residues in two protein chains and had an *R*/*R*_free_ of 24.7%/27.0%. This model was improved through iterative rounds of model building using Coot^39^ and refinement using phenix.refine^38^ resulting in a final *R*/*R*_free_ value of 17.2%/20.9% (Supplementary Table 2). The final model includes 253 residues (amino acids 38–163 in chain A and amino acids 37–163 in chain B), 1 Cu ion, 126 water molecules, and a single sulfate ion. The Ramachandran plot indicates that 99.2% of residues are in favored regions with the remaining 0.8% in allowed regions. The Molprobity^40^ score is 1.29 (99th percentile).

### Construction of a **Δ***pmoD* strain of *Methylosinus trichosporium* OB3b

The *Methylosinus trichosporium* OB3b Δ*pmoD* mutant was generated by chromosomal gene disruption into wild-type *Methylosinus trichosporium* OB3b (a gift from John Lipscomb) using methods described previously^41^. Briefly, a gentamicin resistance gene (*gen*^*R*^) was inserted in the middle of PmoD_*MettrDRAFT_0381*_ via homologous recombination. Primers targeting regions 600 bp upstream and 600 bp downstream from the center of the *pmoD* gene were used to amplify the DNA flanking the inserted *gen*^*R*^. The gentamicin resistance cassette was amplified from vector pFBOH-LIC using primers listed in Supplementary Table 5. The PCR products were assembled into pK18mobsacB_p15a^41^ using Gibson assembly with primers listed in Supplementary Table 5 and transformed into *Escherichia coli* S17-1 ATCC 47055 to produce plasmid pSYR16. This plasmid was then introduced into *Methylosinus trichosporium* OB3b via conjugation with *E. coli* S17-1 cells transformed with pSYR16 on NMS mating agar plates (0 μM CuSO_4_). After 2 days, mated cells were plated on NMS selection agar plates (0 μM CuSO_4_) containing kanamycin (25 μg mL^−1^) and nalidixic acid (10 μg mL^−1^). Single colonies from selection plates were picked and streaked onto NMS counter selection plates (0 μM CuSO_4_) containing 2.5% sucrose and gentamicin (10 μg mL^−1^). Single colonies from counter selection plates were then plated and maintained on NMS agar plates (0 μM CuSO_4_) containing gentamicin (10 μg mL^−1^). Genotyping and sequencing were performed to confirm generation of the mutant using the primers listed in Supplementary Table 542. Cells on NMS agar plates were incubated in GasPak plate incubation chambers (BD) at 30 °C and were subjected daily to vacuum and gas-exchange cycles with gas at a 1:1 methane-to-air ratio and 1 L min^−1^ for 3 min.

### Phenotype analysis of *Methylosinus trichosporium* OB3b strains

Wild-type *Methylosinus trichosporium* OB3b and the Δ*pmoD* strain were cultivated as 50 ml cultures in 250 mL flasks in NMS medium (10 mM NaNO_3_, 1 mM K_2_SO_4_, 150 µM MgSO_4_, and 50 µM CaCl_2_, buffered with 3.5 mM phosphate buffer, pH 6.8) supplemented with trace metals solution (40 µM FeSO_4_, 1.6 µM ZnSO_4_, 1 µM H_3_BO_3_, 0.8 µM MnCl_2_, 0.5 µM KI, 0.2 µM Na_2_MoO_4_, and 0.2 µM CoCl_2_) at 30 °C shaking at 300 rpm. The cultures were sealed with gas-tight rubber septa and fed daily with a 1:3 air/methane mixture^42^. For copper-replete growths, the medium was supplemented with 10 μM CuSO_4_, and for Δ*pmoD Methylosinus trichosporium* OB3b, 5 μg mL^−1^ gentamicin was also included in the growth medium. Growth was monitored daily by withdrawing 1 mL aliquots and measuring the optical density of the cultures at 600 nm. After a week, phenotypes were analyzed using the naphthalene assay to assess sMMO activity. A saturating amount of naphthalene was added to 1 mL cell culture, followed by incubation with agitation at 30 °C for 1 h. sMMO activity was assessed qualitatively by the observation of a color change upon addition of 100 µL 1% solution of Fast Blue B salt (Sigma) to each reaction. The optical spectra of the spent media were analyzed for the presence of apo Mbn by the presence of distinct absorbance features at 345 and 390 nm. One milliliter of culture was centrifuged at 8000 × *g* for 10 min at room temperature and the ultraviolet (UV)–visible light spectrum of the supernatant was measured using an Agilent 8453 UV-visible spectrophotometer^10^. To account for growth variability between cultures, the samples for both the naphthalene assay and Mbn assay were adjusted to the same optical density in NMS medium. Cell lysates were prepared and analyzed using sodium dodecyl sulfate–polyacrylamide gel electrophoresis (SDS-PAGE) as follows. A 50 mg cell pellet was resuspended in 85 μL buffer (250 mM NaCl, 25 mM PIPES, pH 7.0). Cells were lysed by the addition of 200 μL Bacterial Protein Extraction Reagent (B-PER) (Thermo Fisher Scientific) supplemented with 350 μg mL^−1^ lysozyme, 35 μg mL^−1^ DNase I, and 2 µL of a 25× stock solution of cOmplete EDTA-free Protease Inhibitor Cocktail (Roche). After nutation at 4 °C for 30 min, the solution was centrifuged for 25 min at 20,000 × *g* at 4 °C, 4× SDS loading dye was added to the clarified lysate (supernatant), and the sample was run on a 15% SDS-PAGE gel.

### Time-dependent copper-responsive qPCR analysis of *pmoD* genes

All time-dependent copper-responsive qPCR experiments were performed in wild-type *Methylosinus trichosporium* OB3b. For these experiments, as in previous studies^10,43^, *Methylosinus trichosporium* OB3b cultures were grown in nitric acid-washed spinner flasks to minimize copper concentrations. Cells were grown in NMS medium buffered with 3.5 mM phosphate buffer, pH 6.8, and amended with trace metals at a final concentration of 50 µM CuSO_4_, 40 µM FeSO_4_, 1.6 µM ZnSO_4_, 1 µM H_3_BO_3_, 0.8 µM MnCl_2_, 0.5 µM KI, 0.2 µM Na_2_MoO_4_, and 0.2 µM CoCl_2_. Cultures were stirred at 200 rpm and sparged continuously with a 1:3 methane/air mixture. Once cultures achieved logarithmic growth (A_600_ = 0.6–1.0), cells were assayed for the production of Mbn and sMMO as proxies for copper starvation. The presence of Mbn was confirmed via absorbance spectroscopy of clarified spent medium and the production of sMMO was confirmed via the colorimetric naphthalene assay.

Once cells achieved logarithmic phase growth and copper starvation was confirmed, time-course experiments were initiated. Cells were harvested prior to the addition of CuSO_4_ to a final concentration of 12.5 µM, as well as at three subsequent timepoints (15 min, 5 h, and 24 h). All experiments were repeated with three distinct biological replicates. At each timepoint, harvested cells were re-analyzed for Mbn production and sMMO production as well as used for RNA isolation.

All experiments involving RNA used RNase-free reagents and supplies. At each timepoint, culture volumes containing approximately 3 × 10^9^ cells were removed from the harvested cells and added to an ice-cold stop solution containing 5% phenol and 95% EtOH, for a final ratio of 10:1.25 cell culture to stop solution. The cells were then centrifuged at 6000 × *g* for 20 min at 4 °C, and the resulting pellets were resuspended in 0.6 mL TE buffer, supplemented with 20 mg lysozyme (Sigma) and 100 µL proteinase K (Qiagen.) The resuspended cells were incubated for 30 min at room temperature on a rocker. After this incubation step, 5 mL of Qiazol (Qiagen) heated to 65 °C was added to each reaction, and RNA purification was performed using an RNeasy Midi kit (Qiagen), following the protocol described in the RNeasy Lipid Handbook. This initial process included an on-column DNase treatment step. All RNA was eluted in RNase-free water and underwent preliminary quantification using a Nanodrop (Thermo). Any remaining genomic DNA was removed using a Turbo DNAfree kit (Life Technologies). The purified RNA was further processed using an isopropanol/ethanol precipitation step and was resuspended in 20 µL RNase-free H_2_O (Life Technologies). The final RNA concentrations were initially determined using a Nanodrop 1000 (Thermo), but these concentrations were validated using a BioAnalyzer (Agilent), which also yielded RNA integrity numbers for all samples. A SuperScript VILO complementary DNA (cDNA) synthesis kit using random hexamer primers (Life Technologies) was used to synthesize cDNA from 1 µg input RNA.

Some primers used in these experiments had been validated previously^10^, including those for the reference genes and for components of the main *mmo* and *pmo* operons; new primers were validated using the same process. In short, primers were designed using the IDT PrimerQuest tool using constraints including an amplicon length between 70 and 150 bp, no possible products in the genome with fewer than 5 mismatches and 1–2 gaps (as identified using the e-PCR tool (NCBI)), 60 °C ≤ *T*_m_ ≤ 64 °C, ∆*G*_homodimer_ ≥ −7.5, and ∆*G*_hairpin_ ≥ −3. qPCR reactions were carried out in white 384-well PCR plates (Bio-Rad), and each reaction contained 1.2 µL SYBR GreenER Express Universal Master Mix (Life Technologies) and 1.2 µL containing varying amounts of cDNA or RNA (1 ng in standard reactions, fourfold dilutions from 2 ng to 488 fg in primer efficiency reactions), water, and 90 nL pooled forward and reverse primers (5 µM each). Each reaction was run with three technical replicates, and no-template controls and no-reverse-transcriptase controls were also included for every primer/sample. Once prepared, qPCR reactions were incubated at 4 °C for 1 h, centrifuged briefly, and then loaded on a CFX384 (Bio-Rad) thermocycler.

qPCR reactions required an initial 2 min UDG (uracil-DNA glycosylase) inactivation step at 50 °C; this step was followed by a 2 min heating step at 95 °C. These steps were followed by 40 cycles at 95 °C for 15 s and 60 °C for 1 m; plate reading occurred during the combined annealing and extension steps. After the completion of 40 cycles, samples were allowed to anneal at 50 °C for 1 min, and melt curves were then obtained by performing a 5 s plate-reading step at 0.5 °C intervals from 50 °C to 95 °C. Initial Cq values were determined in the Bio-Rad CFX Manager software using regression for each trace. Wells with no Cq value (where replicates had Cq values), a Cq value 10 greater or less than the other technical replicates, or with a melting curve not corresponding to the melting curve peak of the correct product were removed from consideration. The size of all qPCR products and the specificity of all qPCR reactions were confirmed by loading 5 µL reactions onto a 4% NuSieve 3:1 agarose gel (Lonza) in TBE (Supplementary Fig. 5a).

While initial data screening for primer efficiency and gene expression levels were both analyzed in CFX Manager (Bio-Rad), further analysis was performed in qBase^PLUS^^44,45^, including calculation of primer efficiency (Supplementary Data 1) and calculation of normalized relative quantity (NRQ) values for all genes (Supplementary Fig. 5), along with additional statistical analyses (Supplementary Data 1). Hierarchical clustering of log_10_-transformed NRQ data was performed using Cluster 3.0^46^, and these data were used to generate dendrograms and heatmaps in JavaTreeView^47^.

### Proteomics analysis

Membrane-bound pMMO samples from *Methylocystis* sp. strain Rockwell (ATCC), *Methylococcus capsulatus* (Bath) (ATCC), *Methylomicrobium alcaliphilum* 20Z (a gift from Marina Kalyuzhnaya), and *Methylomicrobium buryatense* 5GB1C (a gift from Mary Lidstrom) were prepared from cells grown in copper-replete medium. For *Methylocystis* sp. strain Rockwell, as in previous studies^48^, the growth medium consisted of an adapted NMS-based salt solution consisting of 0.08% w/v KNO_3_, 0.02% w/v K_2_SO_4_, 0.003% w/v MgSO_4_∙7H_2_O, and 0.00007% w/v CaCl_2_∙2H_2_O buffered with 3.9 mM phosphate buffer, pH 7.0, and supplemented by a trace metal solution consisting of 50 µM CuSO_4_, 40 µM FeSO_4_, 2 µM ZnSO_4_, 2 µM H_3_BO_3_, 1.6 µM MnCl_2_, 1 µM KI, 0.4 µM Na_2_MoO_4_, and 0.4 µM CoCl_2_. Cells were grown in 12 L fermentations at 30 °C and 300 rpm with gas sparging at a 3:1 air-to-methane ratio. Once an A_600_ = 5.0–8.0 was reached, cells were harvested by centrifugation at 5–7000 × *g* for 10 min. Cells were washed 1–3 times with a buffer consisting of 25 mM PIPES, pH 6.8, before freezing in liquid nitrogen. For *Methylococcus capsulatus* (Bath), as in previous growths^48^, the growth medium consisted of NMS solution which contains 0.2% w/v KNO_3_, 0.1% w/v MgSO_4_∙7H_2_O, and 0.001% CaCl_2_∙7H_2_O, along with 3.9 mM phosphate buffer, pH 6.8. This medium was supplemented with trace metals, including 50 µM CuSO_4_, 80 µM NaFe(III)EDTA, 1 µM Na_2_MoO_4_, and a trace metal solution composed of 0.6 µM Na_2_•EDTA, 0.4 µM FeSO_4_•7H_2_O, 0.02 µM ZnSO_4_•7H_2_O, 0.008 µM MnCl_2_•4H_2_O, 0.24 µM H_3_BO_3_, 0.042 µM CoCl_2_•6H_2_O, 0.004 µM NiCl_2_•6H_2_O, and 0.006 µM Na_2_MoO_4_•2H_2_O. Cells were grown in 12 L fermentations at 300 rpm and 45 °C with gas sparging at a 4:1 air/methane ratio. Cells were harvested as described for *Methylocystis* sp. Rockwell. As in previous studies^49^, *Methylomicrobium alcaliphilum* 20Z was grown in modified NMS (NMSA) medium amended with 2.3 mM phosphate buffer at pH 7.2, 50 mM carbonate buffer pH 9.5, 500 mM NaCl, 40 µM CuSO_4_ and a trace metal solution containing 0.3 µM Na_2_•EDTA, 0.2 µM FeSO_4_•7H_2_O, 0.009 µM ZnSO_4_•7H_2_O, 0.004 µM MnCl_2_•4H_2_O, 0.1 µM H_3_BO_3_, 0.02 µM CoCl_2_•6H_2_O, 0.002 µM NiCl_2_•6H_2_O, and 0.003 µM Na_2_MoO_4_•2H_2_O. Cells were grown in 12 L fermentation volumes as described for *Methylocystis* sp. Rockwell. Cells were harvested at A_600_ of 8–10, centrifuged at 8000 × *g* for 30 min, and flash frozen in liquid nitrogen. As in previous studies^50,51^, *Methylomicrobium buryatense* 5GB1C was grown in modified NMSA medium amended with 2.3 mM phosphate buffer, pH 6.8, 50 mM carbonate buffer, pH 9.5, 130 mM NaCl, 40 µM CuSO_4_, and a trace metals solution including 5.4 µM Na_2_•EDTA, 14.4 µM FeSO_4_•7H_2_O, 5.5 µM ZnSO_4_•7H_2_O, 0.3 µM MnCl_2_•4H_2_O, 1 µM H_3_BO_3_, 1.7 µM CoCl_2_•6H_2_O, 0.2 µM NiCl_2_•6H_2_O, and 0.4 µM Na_2_MoO_4_•2H_2_O. Growth and harvest were performed as described for *Methylomicrobium alcaliphilum* 20Z.

To purify pMMO^48,51,52^, frozen cells were thawed in a lysis buffer consisting of 25 mM PIPES, pH 7.2 and  250 mM NaCl, except for *Methylomicrobium alcaliphilum* 20Z which required an alternate lysis buffer consisting of 25 mM PIPES, pH 7.2 and 500 mM NaCl. Cells were lysed via sonication, and cell debris was removed via centrifugation at 12,000 × *g* for 1 h at 4 °C. The supernatant was harvested, and membranes were pelleted at 100,000 × *g* for 1 h at 4 °C and flash frozen in liquid nitrogen. pMMO was solubilized from the membrane using 1.2 mg *n*-dodecyl-β-d-maltopyranoside (DDM) (Anatrace) per 1 mg of crude protein for 1 h at 4 °C. The membrane was pelleted via centrifugation at 100,000 × *g* for 30 min at 4 °C, and the supernatant containing the detergent-bound, solubilized pMMO was collected for further purification. *Methylococcus capsulatus* (Bath) pMMO was purified using a Superdex 200 size exclusion chromatography column (GE Healthcare) equilibrated with 25 mM PIPES, pH 7.3, 250 mM NaCl, and 0.02% DDM. pMMOs from *Methylocystis* sp. strain Rockwell, *Methylomicrobium alcaliphilum* 20Z, and *Methylomicrobium buryatense* 5GB1C were purified using a 15Q anion exchange chromatography column (GE Healthcare) with a 50–800 mM NaCl gradient in 25 mM PIPES, pH 7.3, 0.02% DDM. pMMO-containing fractions were concentrated and buffer exchanged into 25 mM PIPES, pH 7.3, 250 mM NaCl, 0.02% DDM using an Amicon centrifugal concentrator (Millipore) with a 100 kDa MWCO.

Proteins were separated via reverse-phase HPLC on an Agilent 1100 HPLC using a 214TP54 analytical C4 column (Grace Vydac: 5 μm, 250 × 4.6 mm i.d.). Approximately 100 µL pMMO (at a concentration of 100 µM) was loaded onto the column, and the protein was eluted using a gradient from 100% A (63.75% formic acid, 10% acetonitrile, 5% *i*-PrOH) to 100% B (70% formic acid, 30% *i*-PrOH) over 60 min at 0.5 mL min^−1^. Then, 100 μL of approximately 100 μM protein (concentration calculated per pMMO protomer) was injected with each run. Elution fractions were collected every 0.25 mL. Samples were then diluted 1:8 with water and digested with 2 μg pepsin at 37 °C overnight (Promega). Samples were heated at 95 °C for 10 min to inactivate pepsin.

Copper-replete *Methylosinus trichosporium* OB3b was grown as described above. Cells were harvested near the late stages of logarithmic growth (an optical density at 600 nm of ~1). Whole cell lysate from *Methylosinus trichosporium* OB3b cells was obtained using an SDS lysis buffer containing 4% SDS, 100 mM HEPES, pH 7.2, 10 mM DTT, and EDTA-free cOmplete protease inhibitors (Roche) at a final volume of 1 tablet per 50 mL lysis buffer. Proteins were precipitated with 8 volumes of cold acetone and one volume of trichloroacetic acid overnight at −20 °C. After washing the pellet with ice-cold acetone, resulting proteins were resuspended in 50 μL deionized 8 M urea in 100 mM ammonium bicarbonate, pH 7.8, reduced with 10 mM DTT at 50 °C for 30 min, and cysteines were alkylated with 35 mM iodoacetamide in the dark for 30 min. The solution was then diluted to <2 M urea (final concentration) and 1 μg porcine trypsin (Thermo) was added prior to overnight incubation at 37 °C with shaking.

For both samples, the resulting peptides were desalted using solid phase extraction on a Pierce C18 Spin column and eluted in 80 μL of 80% acetonitrile in 0.1% formic acid. After lyophilization, peptides were reconstituted with 5% acetonitrile in 0.1% formic acid and injected onto a trap column (150 μm i.d. × 3 cm) coupled with a nanobore analytical column (75 μm i.d. × 15 cm, both ReproSil C18aq, 3 µm). Samples were separated using a linear gradient of solvent A (95% water, 5% acetonitrile, 0.1% formic acid) and solvent B (5% water, 95% acetonitrile, 0.1% formic acid) over 60 min. Mass spectrometry (MS) data were obtained on a Velos Orbitrap Elite (Thermo) mass spectrometer. Data were searched using Mascot (Matrix Science) 2.5 against the SwissProt database and results were reported at 1% false discovery rate in Scaffold 4.5 (Proteome Software).

### Bioinformatics analyses

There are no pre-existing protein families to which PmoD sequences belong, and *pmoD* genes are commonly unannotated or are annotated solely as a hypothetical membrane protein. Thus, the PmoD_*MettrDRAFT_0381*_ sequence was used as an initial seed for a protein BLAST search against the JGI/IMG genome database. The most divergent result was used as the seed for a second BLAST round, and this was repeated until no additional PmoD sequences were obtained. The amino acid sequences of these genes and their associated metadata were downloaded on 10 October 2017.

All putative PmoD sequences were aligned via MAFFT (L-INS-I mode)^53^. After alignment, sequences were trimmed. Truncated sequences with less than 150 amino acids (aa) were removed from the dataset, as were sequences with more than 250 aa that did not contain the two characteristic cysteines along with an N-terminal signal peptide sequence and a C-terminal transmembrane sequence. The trimmed dataset included 860 putative PmoD sequences, of which 98.5% were found in species encoding an enzyme homologous to pMMO and AMO. The metadata file was updated to remove trimmed sequences (Supplementary Data 2), and the trimmed dataset (Supplementary Data 3) was realigned via MAFFT and used to establish a HMM (Supplementary Data 4) via HMMER 3.1^54^. PmoD subgroups were aligned against the profile HMM using HMMalign (Supplementary Figure 7) to visualize differences in subgroup sequence conservation.

A sequence similarity network was constructed using the EFI-EST tool^55^, with an *E*-value cutoff of 21 and condensation of identical sequences into a single node. The resulting network (Supplementary Data 5) was visualized in Cytoscape 3.5.1^56^. As in previous studies^30^, additional metadata was added for each gene for visualization within the context of the sequence similarity network. This included genus (from which subgroups of methane- and ammonia-oxidizing bacteria were identified) and conservation of specific residues of interest using the alignment generated via hmmalign. For genome neighborhood analysis, hierarchical clustering of *pmoD* genes against traits (presence of genes annotated with specific PFAM and TIGRFAM families within 5 genes up- and downstream from *pmoD*-specific metal binding motifs) was carried out as described previously. The resulting dataset (Supplementary Data 2) was examined to establish 20 main operon groups (Supplementary Figure 8).

To analyze the co-occurrence of specific copper-related genes with CuMMO-encoding operons, lists of genomes containing genes corresponding to specific protein families were generated by analyzing all genomes in the JGI/IMG database. The *a/pmoB* genes (as a proxy for *amo*/*pmo* operons) were identified as belonging to the PF04744 family; sMMO-encoding operons were identified as members of the TIGR04550 family which corresponds to *mmoD* genes, not generally found in non-sMMO soluble diiron monooxygenase operons; *mbn* operons were identified via BLAST and manually curated using an *E*-value cutoff of 20^57^ using the *Methylosinus trichosporium* OB3b *mbnB* gene (MettrDRAFT_3422, corrected to remove the unconserved internal stop codon in the published genome)^10^; MopE/CorA homologs were identified using BLAST (against *Methylomicrobium album* BG8 *corA* gene (MetalDRAFT_3218) and *Methylococcus capsulatus* Bath *mopE* gene (MCA2589), since the existing profile HMM, which defines family PF11617, matches only a small region of the MopE/CorA sequence and thus lacks specificity beyond the putative metal binding site); *csp1/2* genes were identified as belonging to the TIGR04401 family; *csp3* genes were identified as belonging to the PF03860 family or were identified as one of the top 500 BLAST results for *Methylosinus trichosporium* OB3b *csp3* gene (MettrDRAFT_1424), since some *csp3* genes more closely related to the *Methylosinus trichosporium* OB3b gene were not annotated as DUF326 genes (Supplementary Discussion); *pmoD* genes were identified as described above. Lists of genomes containing each class of gene were extracted from the JGI/IMG gene metadata, and the co-occurrence of genes of various classes in a given genome was quantified.

## Electronic supplementary material

Supplementary Information

Description of Additional Supplementary Files

Supplementary Data 1

Supplementary Data 2

Supplementary Data 3

Supplementary Data 4

Supplementary Data 5

## Data Availability

The coordinates and structure factors for the PmoD_*Met49242_1452*_ structure have been deposited in the Protein Databank with accession code 6CPD. Other data are available from the corresponding author upon reasonable request.
