# Supplementary material for: Characterization of a long overlooked copper protein from methane- and ammonia-oxidizing bacteria
Source: Nat Commun. 2018 Oct 15;9:4276. doi: 10.1038/s41467-018-06681-5 (PMC6189053; doi:10.1038/s41467-018-06681-5)
Supplement: Supplementary file 1 — Supplementary Information [file 41467_2018_6681_MOESM1_ESM.pdf]

Characterization of a long overlooked copper protein from methane- and ammonia-oxidizing bacteria

O. S. Fisher, et al.

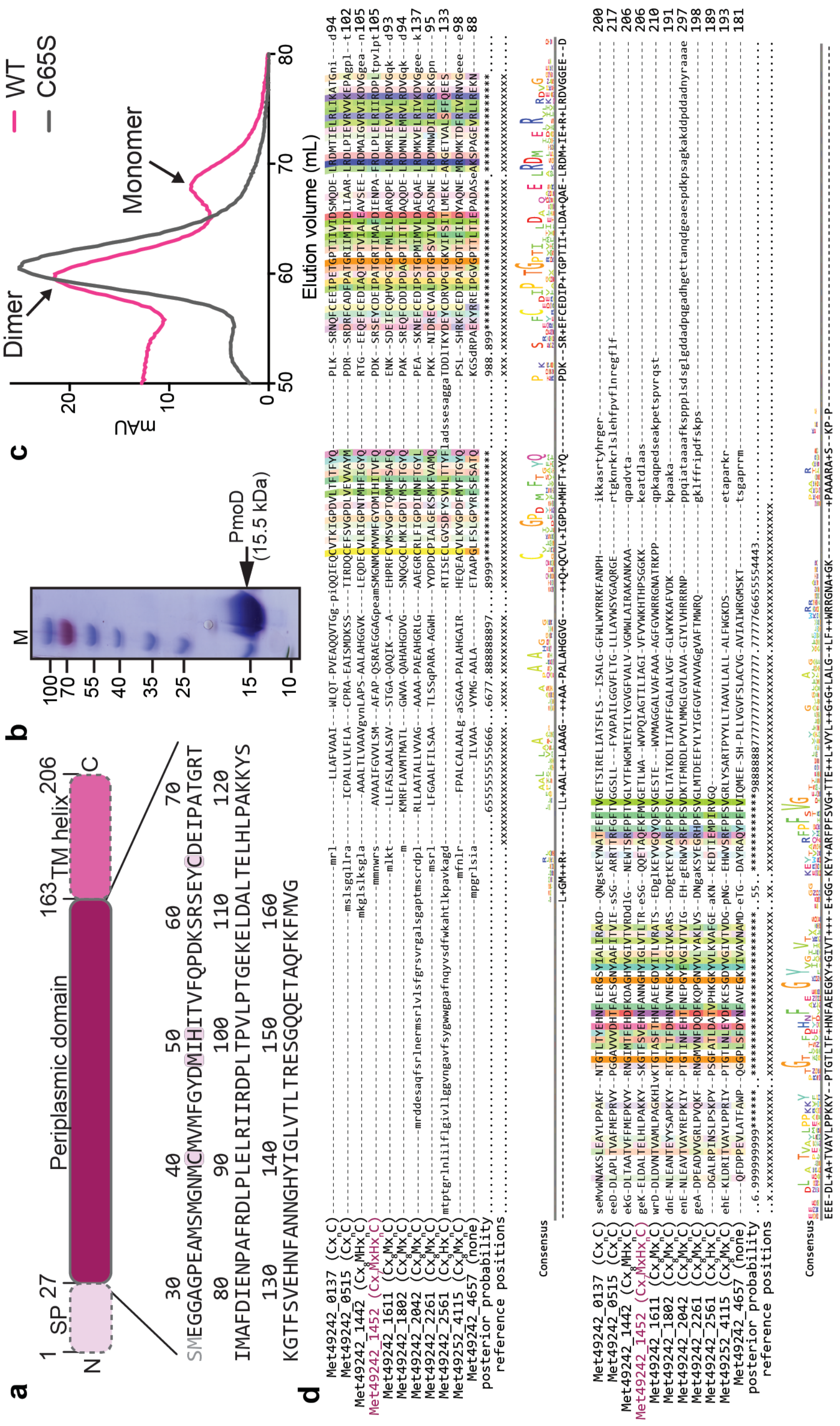

Supplementary Figure 1 | PmoD$_{Met49242_1452}$ periplasmic domain. a, Schematic depiction of PmoD sequence, with signal peptide (SP), periplasmic domain, and transmembrane (TM) helix indicated. The amino acid sequence of the periplasmic domain is shown on the bottom. The vector-derived residues in the construct are in grey, the two conserved cysteines in yellow, and the methionine and histidine residues identified as Cu_A site ligands are in bold. **b,** SDS-PAGE gel showing the purified PmoD$_{Met49242_1452}$ periplasmic domain. **c,** Size exclusion chromatogram of copper-loaded PmoD$_{Met49242_1452}$. The WT protein elutes as two species, a dimer that exhibits the characteristic color and spectral features of the Cu_A site and a monomer that does not. The Cys65Ser variant elutes primarily as a dimer exhibiting the characteristic color and features of the Cu_A site. **d,** *Methylocystis* sp. Rockwell has eleven PmoD homologues. These are not recent gene duplications: significant divergence in protein sequence is observed, with six different variations on the potential copper binding motifs among the various homologues.

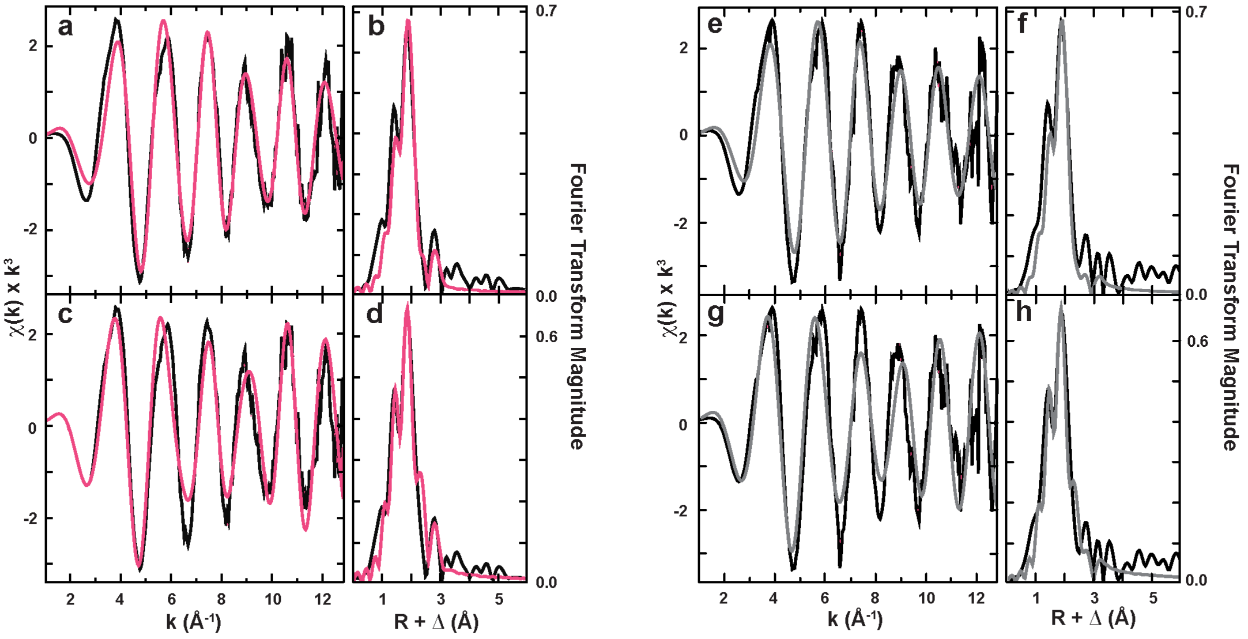

Supplementary Figure 2 | Cu EXAFS data and fits for wildtype and C65S PmoD$_{Met49242_1452}$. **a** and **b,** Cu EXAFS data and fit for the best fit simulation for wildtype PmoD$_{Met49242_1452}$ using the standard conservative approach that follows rules governing both spectral resolution relative to acceptable intra-ligand scattering interaction bond lengths and acceptable bond lengths. **c** and **d,** Cu EXAFS data and fit for the best fit simulation for wildtype PmoD$_{Met49242_1452}$ including a constrained Cu-Cu vector. EXAFS spectra are shown in panels **a** and **c**, and the Fourier transforms of the Cu EXAFS are shown in panels **b** and **d**. **e** and **f,** Cu EXAFS data and fit for the best fit simulation for C65S PmoD$_{Met49242_1452}$ using the standard conservative approach. **g** and **h,** Cu EXAFS data and fit for the best fit simulation for C65S PmoD$_{Met49242_1452}$ including a constrained Cu-Cu vector. EXAFS spectra are shown in panels **e** and **g**, and the Fourier transforms of the Cu EXAFS are shown in panels **f** and **h**. For all panels, raw unfiltered data are shown in black, and the best fit simulations are shown in either pink (wildtype) or grey (C65S). EXAFS were fit over a *k*-range of 1.0-12.85 $Å^{-1}$.

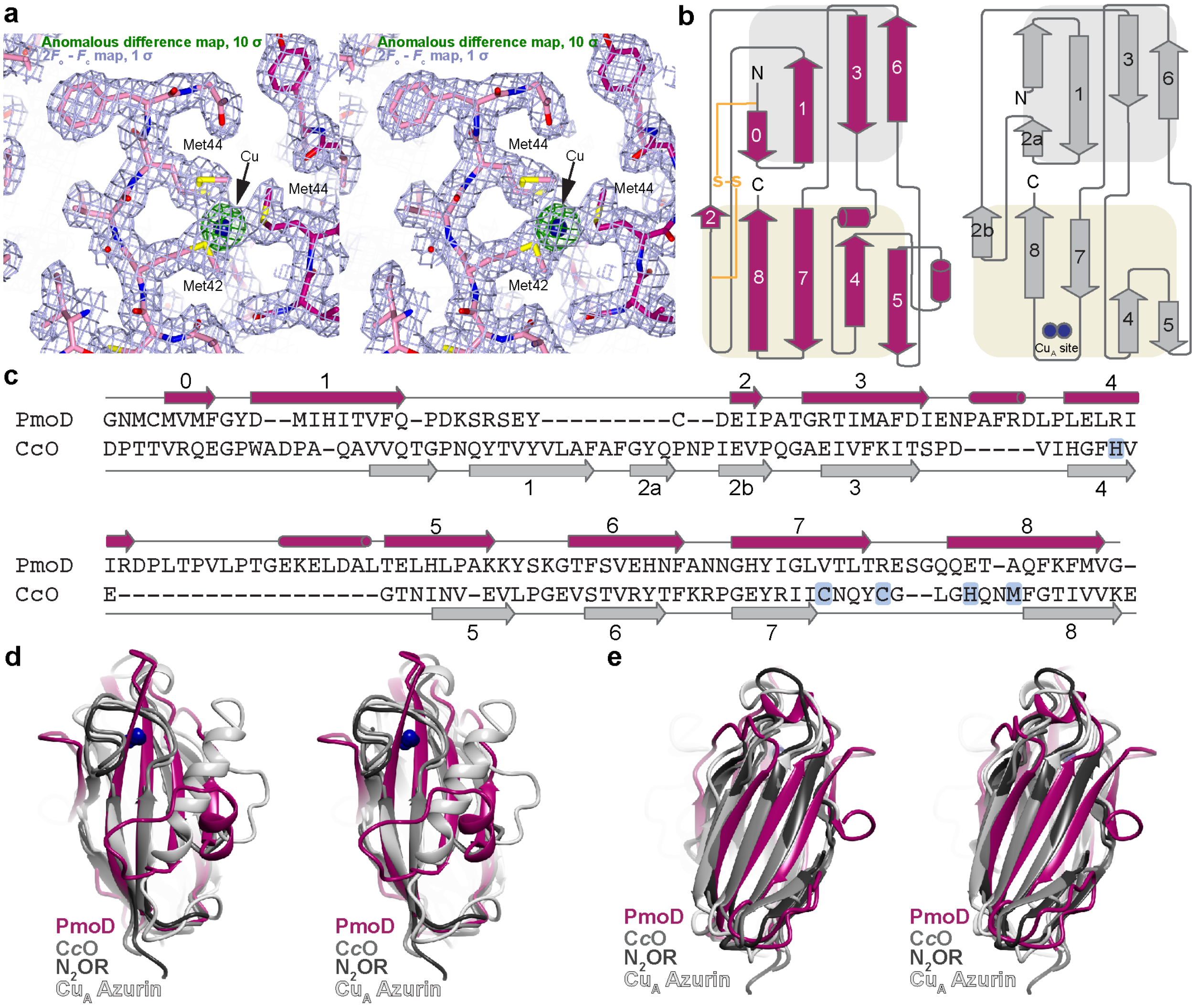

Supplementary Figure 3 | Structural analysis of PmoD$_{Met49242_1452}$ periplasmic domain. a, Stereoview of the electron density surrounding the Cu site in the crystal structure. The $2F_o - F_c$ map is shown in blue mesh contoured at 1σ, and the anomalous difference map in orange mesh contoured at 10σ. **b,** Topology diagrams of the PmoD$_{Met49242_1452}$ structure (left) and the *T. thermophilus* C*c*O Cu$_A$-containing domain (right). The location of the Cu$_A$ site is shown as blue circles. **c**, Sequence alignment between PmoD$_{Met49242_1452}$ and the C*c*O Cu$_A$ domain. Secondary structure elements are shown above the sequence in magenta for PmoD$_{Met49242_1452}$ and below the sequence in grey for C*c*O. The residues that comprise the Cu$_A$ binding motif in C*c*O are highlighted in blue. **d,** Stereoview of a superposition of the PmoD$_{Met49242_1452}$ structure (magenta) with the structures of *T. thermophilus* C*c*O (PDB: 2CUA, grey, residues 70-168 of chain A), *Paracoccus denitrificans* N_2OR (PDB: 1FWX, dark grey, residues 479-581 of Chain A), and *Pseudomonas aeruginosa* engineered Cu$_A$ azurin (PDB: 1CC3, white; chain A). **e,** Stereoview of the same superposition shown in **d**, rotated by 180 degrees.

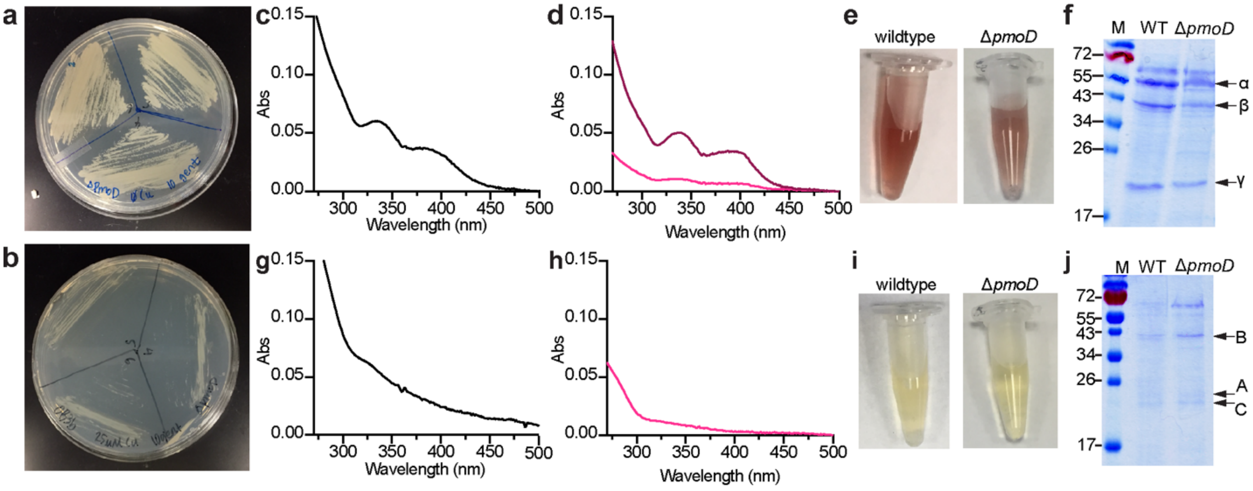

Supplementary Figure 4 | Phenotypic comparison of wildtype and Δ*pmoD* strains of *Methylosinus trichosporium* OB3b. **a,** Δ*pmoD Methylosinus trichosporium* OB3b grown for 1 week on NMS agar that was not supplemented with $CuSO_4$. **b,** Δ*pmoD Methylosinus trichosporium* OB3b grown for 1 week on NMS agar supplemented with 25 μM $CuSO_4$. The cell density is much lower than that in panel **a**. **c,** Optical spectrum of spent media from wildtype strain from cells at $OD_{600} = 1$ grown under copper starved conditions. **d,** Optical spectra of spent media from Δ*pmoD* strain under copper starved conditions. Dark purple spectrum is from cells at $OD_{600} = 1$; pink spectrum is from cells diluted to $OD_{600} = 0.2$. Spectral features at 340 nm and 390 nm associated with Mbn are observed in both samples. **e,** Representative results of naphthalene assay conducted on wildtype (left) and Δ*pmoD* (right) grown under copper starved conditions. The purple color indicates sMMO activity. **f,** Coomassie stained gels of clarified cell lysates grown under copper starved conditions. Bands corresponding to the expected molecular weights of the sMMO subunits are indicated. **g,** Optical spectrum of spent media from wildtype strain grown in 10 μM $CuSO_4$ from cells at $OD_{600} = 1$. **h,** Optical spectrum of spent media from Δ*pmoD* strain grown with 10 μM $CuSO_4$. Cells were at $OD_{600} = 0.2$. The characteristic feature associated with Mbn production are absent in both copper-replete samples. **i,** Representative results of naphthalene assay conducted on wildtype (left) and Δ*pmoD* (right) grown in 10 μM $CuSO_4$. No sMMO activity is observed. **j,** Coomassie stained gels of clarified cell lysates grown with 10 μM $CuSO_4$. Bands corresponding to the expected molecular weights of the pMMO subunits are indicated.

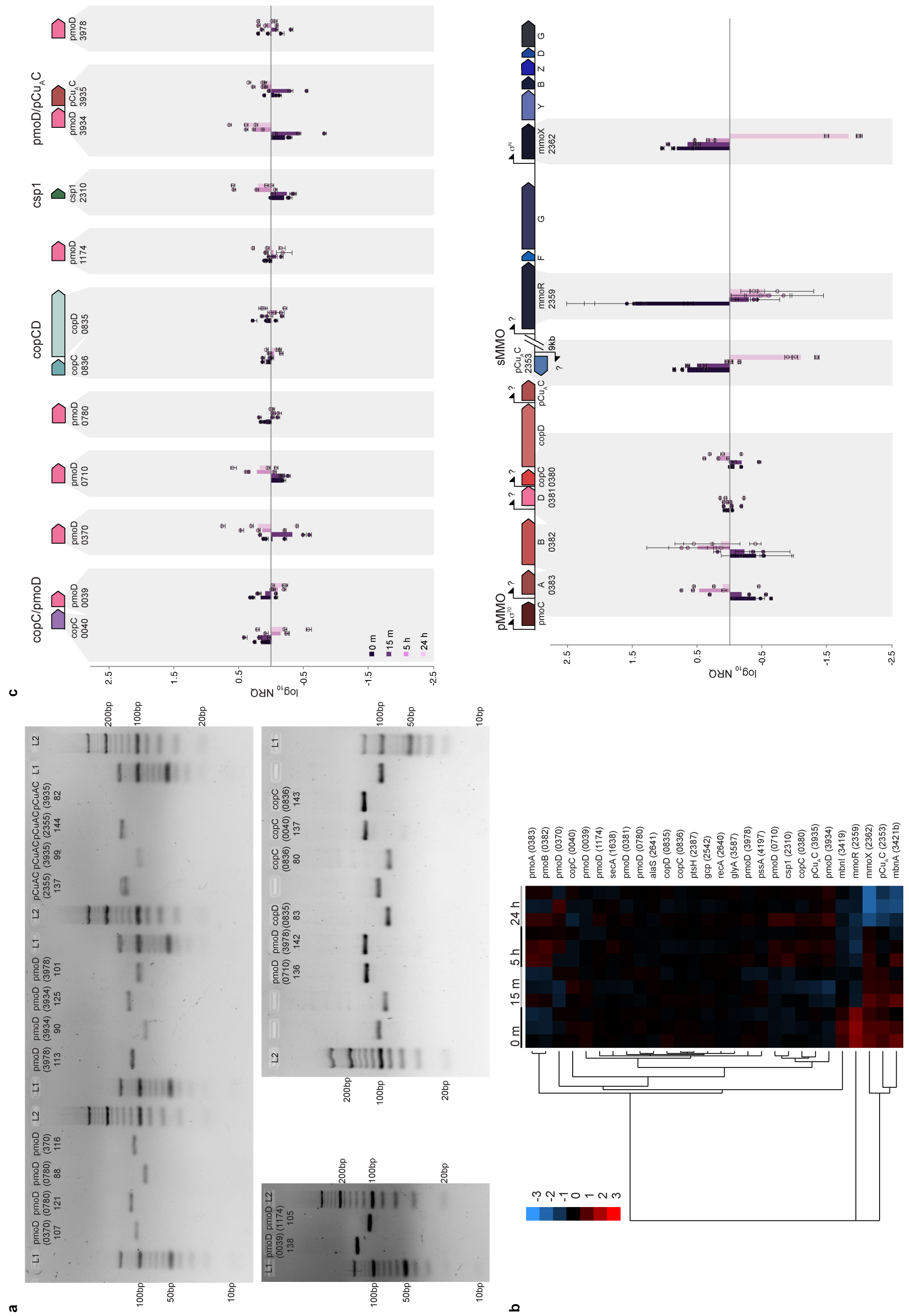

Supplementary Figure 5 | Copper-dependent *pmoD* regulation over time. **a,** Visualization of qPCR products on a 4% NuSieve agarose gel. Amplicon size is provided beneath the gene name and the locus tag (MettrDRAFT_NNNN). Lanes without labels are for primers not discussed in this manuscript; not all primers depicted were used in the final experiments (generally due to amplification efficiency). O'Range Ruler 10 bp ("L1") and 20 bp ("L2") ladders were used to compare amplicon sizes. **b,** A heatmap of gene expression trends observed for genes of interest in *Methylosinus trichosporium* OB3b. Downregulation is indicated in blue and upregulation in red, and values are $\log_{10}$-transformed normalized relative quantities. Genes were hierarchically clustered on the basis of their expression data using Euclidian distance and centroid linkage, as described previously[6,11]. Experiments were performed with three biological replicates at four timepoints (0 min at 0 µM $CuSO_4$, 15 min at 12.5 µM $CuSO_4$, 5 h at 12.5 µM $CuSO_4$, and 24 h at 12.5 µM $CuSO_4$) for wildtype *Methylosinus trichosporium* OB3b. **c,** Copper-responsive gene regulation for wildtype *Methylosinus trichosporium* OB3b is shown separately for all three biological replicates at all four timepoints. Error bars represent standard error.

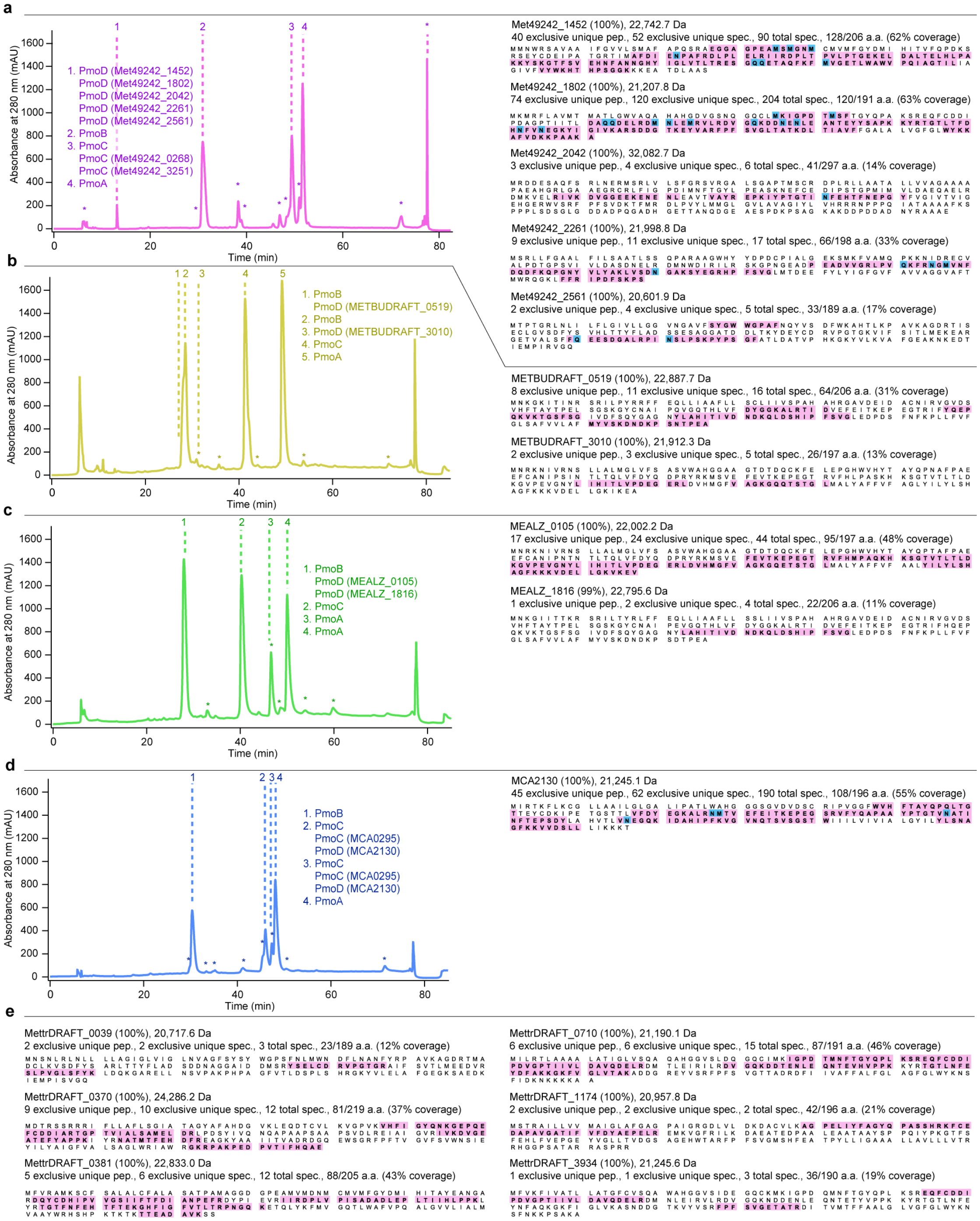

Supplementary Figure 6 | pMMO is the primary component of solubilized pMMO samples, but PmoD peptides are detected in all pMMO samples. a, C4 elution trace for solubilized pMMO from *Methylocystis* sp. Rockwell, a Type II methanotroph. Elution points for pMMO subunits, non-pMMO associated PmoCs and PmoDs are indicated. Locus tags are (Met49242_NNNN), and peptide coverage for the five individually observed PmoDs is depicted to the right of the elution trace. Observed peptides are highlighted in pink; residues with commonly observed mass shifts (generally artifacts related to sample preparation, including methionine oxidation and asparagine and glutamine deamidation) are highlighted in blue. **b,** C4 elution trace for solubilized pMMO from *Methylomicrobium buryatense* 5GB1C, a Type I methanotroph. pMMO and PmoD elution points are highlighted on the chromatogram, and PmoD sequences (and peptide coverage) for both observed PmoDs are presented to the right of the elution trace. **c,** C4 elution trace for solubilized pMMO from *Methylomicrobium alcaliphilum* 20Z, a Type I methanotroph. pMMO and PmoD elution points are highlighted on the chromatogram, and observed peptides are highlighted in pink in the PmoD sequences to the right. **d,** C4 elution trace for solubilized pMMO from *Methylococcus capsulatus* Bath, a Type X methanotroph. The elution points for pMMO subunits, non-pMMO associated PmoCs, and PmoDs are highlighted on the chromatogram, and observed peptides are highlighted in the sole PmoD sequence to the right. **e,** *Methylosinus trichosporium* OB3b whole cell lysate from pMMO-producing cells contains several PmoDs; observed peptides (from six of eight genomically encoded PmoDs) are highlighted in pink.

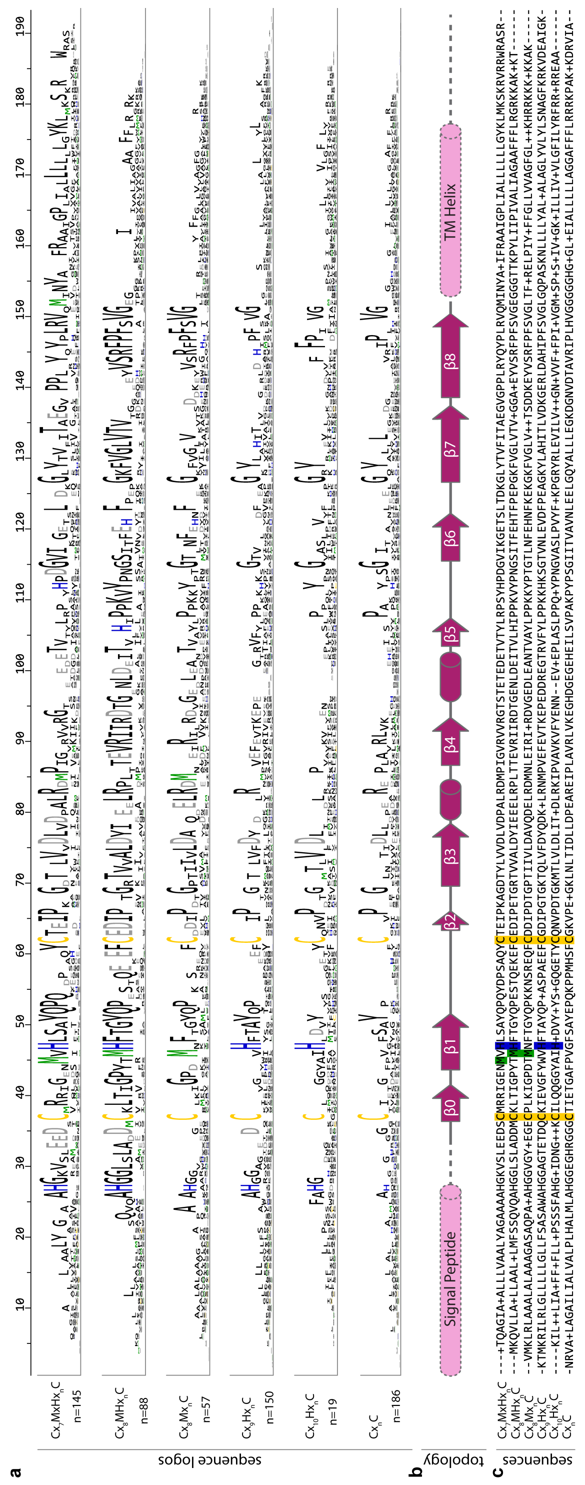

Supplementary Figure 7 | Alignment of PmoD subgroups against the PmoD Hidden Markov Model. a, Sequence logos of PmoD subgroups after alignment against the PmoD HMM. Residues that do not match a position in the profile HMM have been removed for clarity. Only two sequences in this dataset lack the two cysteines entirely, and only two sequences have a Cx_7Mx_nC copper site; none of those sequences are depicted here. Logos were generated using sequences represented as nodes in the EFI network to avoid overrepresentation of sequences from closely related species. Metal-binding residues are highlighted in yellow (cysteine), blue (histidine), green (methionine), and grey (aspartate and glutamate). **b**, Secondary structure of PmoD based on the structure determined of the *pmo* operon homologue from *Methylocystis* sp. Rockwell, presented for reference against the PmoD sequences. Predicted secondary structure elements outside the boundaries of the construct used for structure determination are indicated by dashed lines. **c**, Consensus sequences of the PmoD subgroups, aligned and with the metal-binding residues highlighted.

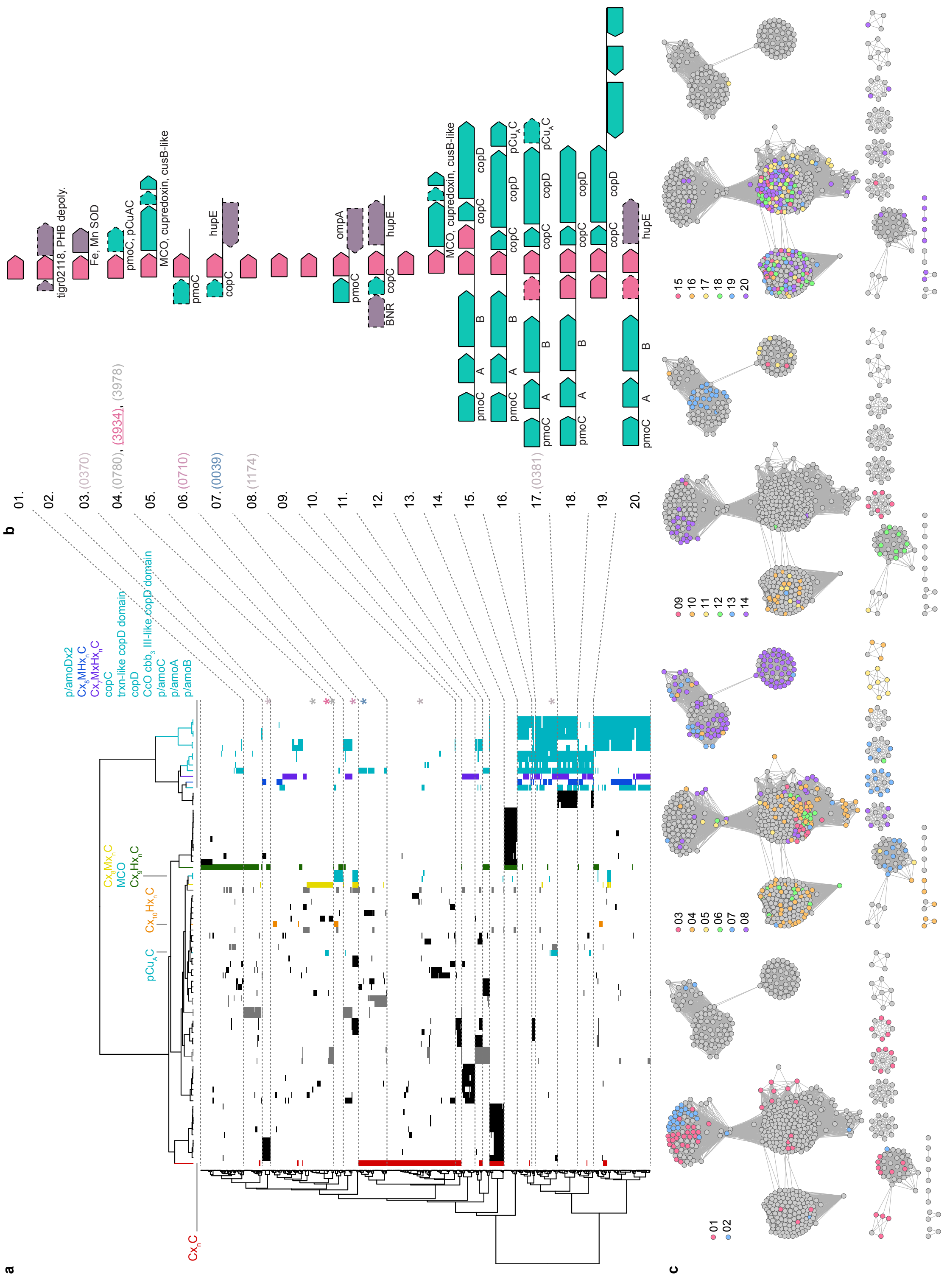

Supplementary Figure 8 | Hierarchical clustering of *pmoD* genes according to traits found in their genomic neighborhood. a, Heatmap establishing correlations between specific traits and specific subsets of *pmoD* genes. **b,** Top 20 typical *pmoD* genomic neighborhoods. Locus tag codes for the eight *Methylosinus trichosporium* OB3b *pmoDs* are immediately adjacent to the operon group numbers. **c,** Sequence similarity networks for PmoD, used to visualize the distribution of specific operon families.

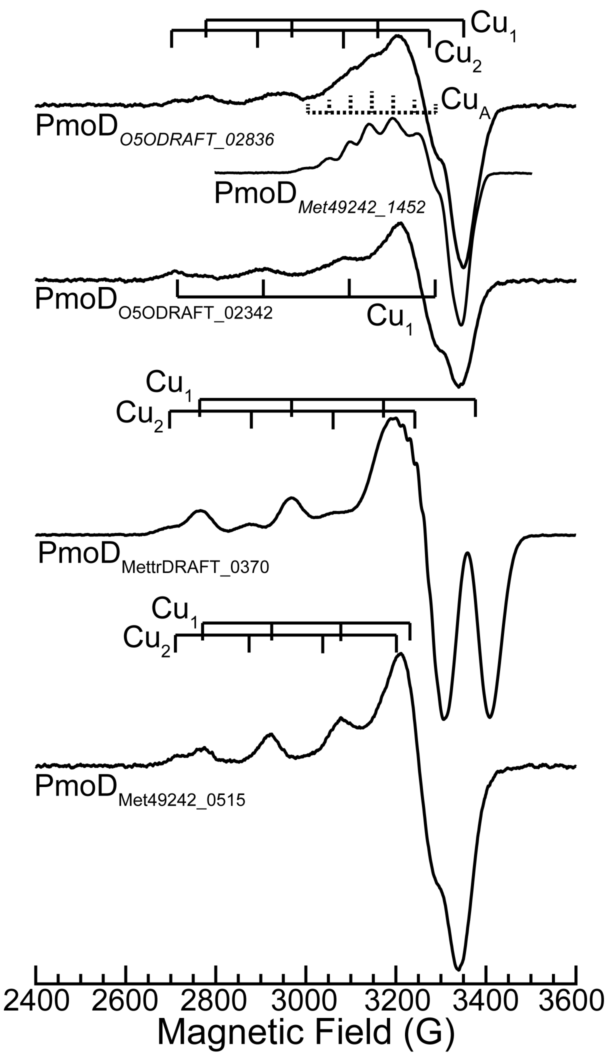

Supplementary Figure 9 | X-band EPR spectra of the copper-loaded PmoD homologues depicted in Fig. 4e. Brackets are centered at $g_{\|}$ and depict the Cu hyperfine splitting $A_{\|}$. $g_{\|}$- and $A_{\|}$-values are provided in Supplementary Table 4. All PmoD EPR spectra except $\text{PmoD}_{\textit{Met49242\_1452}}$ exhibit type 2 Cu(II) resonance; $\text{PmoD}_{\textit{Met49242\_1452}}$ exhibits exclusively Cu_A resonance. $\text{PmoD}_{\textit{O5ODRAFT\_02836}}$ features type 2 Cu(II) and Cu_A resonance, although the Cu_A resonance is mostly unresolved on top of the overlapping Cu_1(II) and Cu_2(II) resonances. The $\text{PmoD}_{\textit{Met49242\_1452}}$ spectrum is presented as an exemplar Cu_A EPR signal. Conditions: 9.364-9.365 GHz microwave frequency, 12.5 G modulation amplitude, 320 ms time constant, 90 s per scan, temperature 20 K, except $\text{PmoD}_{\textit{Met49242\_1452}}$ (conditions listed in Fig. 1). All spectra were background subtracted and intensities were normalized to protein concentration, except for that of $\text{PmoD}_{\textit{Met49242\_1452}}$.

Supplementary Table 1 | Summary of Cu EXAFS simulations.

	Nearest Neighbor Ligand Environment *								Long-Range Ligand Environment*				
**Sample**	**Atom**†	**R(Å)**‡	**CN**§	**σ^2** ‖	**Atom**†	**R(Å)**‡	**CN**§	**σ^2** ‖	**Atom**†	**R(Å)** ‡	**CN**§	**σ^2** ‖	***F'*** ¶
**WT**	**O/N**	**1.98**	**1.0**	**5.86**	**S**	**2.31**	**1.5**	**4.60**	**C**	**3.37**	**1.0**	**2.64**	**0.18**
	O/N	*1.97*	*1.0*	*6.74*	*S*	*2.36*	*2.0*	*7.66*	*C*	*3.38*	*1.0*	*2.18*	*0.33*
	Cu	*2.45*	*0.5*	*2.50*									
	O/N	*1.99*	*1.0*	*5.67*	*S*	*2.35*	*2.0*	*5.76*	*C*	*3.37*	*1.0*	*1.55*	*0.31*
	Cu	*2.43*	*0.5*	*2.50*									
	O/N	***1.99***	***1.0***	***5.13***	***S***	***2.35***	***2.0***	***4.41***	***C***	***3.37***	***1.0***	***1.26***	***0.29***
	Cu	***2.41***	***0.5***	***2.50***									
	O/N	*1.98*	*1.0*	*4.92*	*S*	*2.34*	*2.0*	*3.48*	*C*	*3.37*	*1.0*	*1.35*	*0.29*
	Cu	*2.39*	*0.5*	*2.50*									
**C65S**	**O/N**	**1.98**	**1.0**	**5.13**	**S**	**2.32**	**1.5**	**4.59**	**C**	**3.46**	**0.5**	**2.03**	**0.31**
	O/N	*2.00*	*1.0*	*5.71*	*S*	*2.36*	*2.0*	*6.62*	*C*	*3.45*	*0.5*	*1.00*	*0.57*
	Cu	*2.45*	*0.5*	*2.50*									
	O/N	*2.00*	*1.0*	*5.01*	*S*	*2.36*	*2.0*	*5.05*	*C*	*3.44*	*0.5*	*1.92*	*0.56*
	Cu	*2.43*	*0.5*	*2.50*									
	O/N	***2.00***	***1.0***	***4.73***	***S***	***2.35***	***2.0***	***3.95***	***C***	***3.44***	***0.5***	***2.76***	***0.53***
	Cu	***2.41***	***0.5***	***2.50***									
	O/N	*1.99*	*1.0*	*4.72*	*S*	*2.34*	*2.0*	*3.20*	*C*	*3.44*	*0.5*	*3.30*	*0.54*
	Cu	*2.39*	*0.5*	*2.50*									

* Independent metal-ligand scattering environment.

† Scattering atoms: C (carbon), N (nitrogen), O (oxygen), S (sulfur), Cu (copper).

‡ Average metal-ligand bond length from two independent samples.

§ Average metal-ligand coordination number from two independent samples.

‖ Average Debye-Waller factor in $Å^2 \times 10^3$ from two independent samples.

¶ Number of degrees of freedom weighted mean square deviation between data and fit.

Best fit using conservative simulation strategy is shown in bold/normal font. Best fit using constrained Cu-Cu vector simulation parameters is shown in bold/italics font; the manually varied but constrained Cu-Cu bond length value is underlined. EXAFS were fit over the *k*-range of 1.0-12.85 $Å^{-1}$, for a spectral resolution of 0.13 Å.

Supplementary Table 2 | Data collection and refinement statistics

	$PmoD_{Met49242\_1452}$
**Data collection**	
Space group	$P\ 3_221$
Cell dimensions	
a, *b*, *c* (Å)	83.3, 83.3, 71.0
α, β, γ (°)	90, 90 120
Resolution (Å)	50.0-1.90 (1.97-1.90)*
R_{sym}	8.1 (82.8)*
$I/\sigma I$	37.2 (2.9)*
Completeness (%)	97.2 (82.9)*
Redundancy	27.5 (10.0)*
**Refinement**	
Resolution (Å)	35.9-1.90 (1.93-1.90)*
No. reflections	22042
R_{work} / R_{free} (%)	17.5 / 20.9
No. atoms	
Protein	2006
Ligand/ion	6
Water	126
B-factors ($Å^2$)	
Protein	43.4
Ligand/ion	37.1
Water	44.4
R.m.s. deviations	
Bond lengths (Å)	0.008
Bond angles (°)	1.26

*Values in parentheses are for highest-resolution shell.
Data were collected on 1 crystal.

Supplementary Table 3 | PmoD copper binding stoichiometries

Protein	Cu equiv per protein*
PmoD$_{O5ODRAFT_02836}$ (Cx_7MxHx_nC motif)	1.23 ± 0.11
PmoD$_{O5ODRAFT_02342}$ (Cx_8Mx_nC motif)	0.54 ± 0.13
PmoD$_{MettrDRAFT_0370}$ (Cx_9Hx_nC motif)	1.40 ± 0.18
PmoD$_{Met49242_0515}$ (Cx_nC motif)	1.9 ± 0.02

*Each measurement was performed 3 times on 2 independent samples.

Supplementary Table 4 | PmoD EPR parameters

PmoD	Cu(II) Identity	$g_{\|}$	$A_{\|}$ (MHz)
$PmoD_{O5ODRAFT\_02836}$ (Cx_7MxHx_nC motif)	Cu_1	2.18	585
	Cu_2	2.24	600
	Cu_A*	2.13	140
$PmoD_{O5ODRAFT\_02342}$ (Cx_8Mx_nC motif)	Cu_1	2.23	595
$PmoD_{MettrDRAFT\_0370}$ (Cx_9Hx_nC motif)	Cu_1	2.18	620
	Cu_2	2.25	575
$PmoD_{Met49242\_0515}$ (Cx_nC motif)	Cu_1	2.23	480
	Cu_2	2.26	520

*$PmoD_{O5ODRAFT\_02836}$-Cu_A $g_{\|}$ and $A_{\|}$ values are taken from the $PmoD_{Met49242\_1452}$-Cu_A and may deviate slightly for the $PmoD_{O5ODRAFT\_02836}$-Cu_A, since the $PmoD_{O5ODRAFT\_02836}$-Cu_A $g_{\|}$ and $A_{\|}$ features are mostly unresolved on top of the overlapping Cu_1(II) and Cu_2(II) resonances.

Supplementary Table 5 | Primers used for cloning and sequencing

Target	Template	Forward primer sequence	Reverse primer sequence
pCSG-His plasmid	$PmoD_{Met49242\_1452}$	AAACAGCTATGACATGATTAATTCTCATCGCGCTCTGCTCTTG	ACCCAAGTACCGCCACCTAAAGCTCTATCGGACCGGCACGT
pCSG-His plasmid	$PmoD_{O5ODRAFT\_02836}$	TACTTCCAATCCATGGAGGGCGGGGCCGGAC	TATCCACCTTTACTGTTAGCCCACCATGAATTTGAACAGCTC
pCSG-His plasmid	$PmoD_{Met49242\_0515}$	TACTTCCAATCCATGATCAGCATGGACAAGAGCTCAAC	TATCCACCTTTACTGTTAGCCGACCGTGAAGCCGAATC
pCSG-His plasmid	$PmoD_{O5ODRAFT\_02342}$	TACTTCCAATCCATGCATGGGCGTCTCGGCGC	TATCCACCTTTACTGTTAGTCGACCGAGAAGGGGAAG
pCSG-His plasmid	$PmoD_{MettrDRAFT\_0370}$	TACTTCCAATCCATGCATGACGGCGTGAAGCTCG	TATCCACCTTTACTGTTAGCCGACCGTGAAGGGAAAG
C41S mutagenesis	$PmoD_{Met49242\_1452}$ in pCSG-His	GTCGATGGGGAACATGAGCATGGTCATGTTCGG	CCGAACATGACCATGCTCATGTTCCCCATCGAC
C65S mutagenesis	$PmoD_{Met49242\_1452}$ in pCSG-His	GAATTTCATCGCTATATTCGCTGCGCGATTTGTCC	GGACAAATCGCGCAGCGAATATAGCGATGAAATTC
pmoD (downstream)	*Mst.* OB3b gDNA	AAACAGCTATGACATGATTAATTCTCATCGCGCTCTGCTCTTG	ACCCAAGTACCGCCACCTAAAGCTCTATCGGACCGGCACGT
Gentamicin resistance cassette	pFBOH-LIC	TTAGGTGGCGGTACTTGGGTCGA	AGGACAGAAATGCCTCGACTTCGC
pmoD (upstream)	*Mst.* OB3b gDNA	AGTCGAGGCATTTCTGTCCTTCGGCGGAAGATGGATGATGG	TCCCAGTCACGACGTTGTAAATGTTCGTTCGGGCTATGAAATCA
Mobilization vector	pk18mobsacB	TTACAACGTCGTGACTGGGAAA	TAATCATGTCATAGCTGTTTCCTGTGT
pmoD (for genotyping)	*Mst.* OB3b gDNA (wildtype or Δ*pmoD*)	ATTTCACGACATAGCTCCCGACC	CGAAGGCGCATAGAATTTCTATTGC

Supplementary Discussion

EXAFS

The Cu XANES spectra, measured for both WT and C65S PmoD$_{Met49242_1452}$, are very similar and show features reminiscent of published Cu$_A$ XANES spectra (Figure 1)[1]. Low intensity pre-edge features, centered at ca. 8979.5 eV and observed in both spectra, are consistent with 1s$\rightarrow$3d electronic transitions associated with cupric metal[2]. Edge spectral features, observed at ca. 8983.2 eV and 8987.8 eV (first inflection energies of 8982.5 eV and 8985.3 eV) in both samples, are consistent with the Cu(+1.5)-Cu(+1.5) mixed valent dinuclear centers observed in Cu$_A$ model compounds and in the soluble Cu$_A$ domain of *T. thermophilus* C*c*O[1]. There strong similarity of these spectral features between wildtype and C65S PmoD$_{Met49242_1452}$ indicates that the overall copper structural and electronic ligand environments are very similar between the two samples.

The Cu EXAFS for wildtype and C65S PmoD$_{Met49242_1452}$ are consistent with a nearest neighbor ligand environment constructed by both oxygen/nitrogen and sulfur ligands held within a metal coordination structure that is nearly identical. Simulation analysis following standard conservative fitting protocols that account for 1) spectral resolution to define acceptable inter-ligand environment bond distances[3], 2) acceptable ligand disorder (σ^2) parameters that define acceptable ligand coordination numbers[4] and 3) simulation of "goodness of fit" parameters that measure deviation between theoretical and empirical data weighted for adding additional degrees of freedom to the simulation (*F'*) to justify adding additional ligand environments[3], suggest a Cu-nearest neighbor environment dominated by a Cu-O/N ligand(s) at 1.98 Å and Cu-S ligand(s) at ca. 2.31 Å for both samples (bold non-italics values in Supplementary Table 1). Attempts to fit both data sets with a Cu-Cu vector at a short (2.4-2.5 Å) bond distance provided fits that were of poorer quality (higher *F'* values in the fits) compared to Cu-S ligand environment fits at that

distance, and therefore not justified for inclusion. Spectral resolution for Cu protein XAS is typically > 0.13 Å, indicating only ligand environments outside this distance lower limit are acceptable; this resolution is constrained due to the presence of a Zn edge at the end of the Cu EXAFS, typically present in very low levels from cryostat scattering, that limits Cu useful data to a *k* value of 12.85 Å^{-1}[2].

Given the close XANES spectral similarities to authentic Cu_A models and protein samples[1] and the documented pattern that XAS cannot justifiably deconvolute the Cu-S ligand scattering from a very short Cu-Cu ligand environment due resolution limitations[5], we followed an additional static fitting strategy[21] to try to deconvolute Cu-Cu scattering in our protein samples. In these Cu-Cu static fits, inclusion of a defined Cu-Cu ligand environment was set and not allowed to freely vary in the simulations in the same way the Cu-O/N and Cu-S fits were. However, the Cu-Cu distance was manually incremented through bond distances to determine whether a short Cu-Cu vector could be detected in $PmoD_{Met49242\_1452}$ EXAFS. During this strategy, we ignored violations due to data spectral resolution and bond disorder limitations that would violate the conservative fitting strategy. However, we used the simulation F' values to find the minimum in the potential energy simulation surface to determine which Cu-Cu distance best justifies inclusion of a static Cu-Cu vector. The best Cu-Cu bond distance obtained from these static fits was 2.41 Å for both WT and C65S $PmoD_{Met49242\_1452}$ (bold italics values in Supplementary Table 1). Although static Cu-Cu simulations violate standard conservative protocols, and so should be respected as such, it is significant that a short 2.41 Å Cu-Cu vector consistent with that observed for oxidized Cu_A dinuclear copper centers is observed in both the wildtype and C65S $PmoD_{Met49242\_1452}$ samples. Furthermore, the fact that the valence state and the general XANES spectral features for WT and C65S $PmoD_{Met49242\_1452}$ samples also match

this predicted mixed valent Cu_A coordination environment further suggest a conserved copper coordination environment.

qPCR analysis

No *pmoD* genes display copper-responsive expression changes as marked as those seen in the *mmo* and *mbn* operons. Even some genes that exhibit apparent co-regulation with a neighboring gene and correlation with other copper-responsive genes do not exhibit statistically significant changes in expression after careful correction for multiple testing is applied. A pair of *pmoD/copC* genes (MettrDRAFT_0039-0040) are mildly co-repressed by copper (with 1- and 2-fold decreases seen by 5 h and 24 h after copper addition that nevertheless do not pass the threshold for significance, and some correlation with regulatory changes seen in the *mbn* and *mmo* operons). A *pmoD* gene (MettrDRAFT_0370) adjacent to a gene encoding an iron-manganese superoxide dismutase enzyme is at most mildly (2-fold) up-regulated by 24 h (though it displays little significant correlation with known copper-regulated genes), while a second *pmoD* gene with no co-regulated neighbors (MettrDRAFT_0710) is up-regulated 3-fold by 5 h. By contrast, another *pmoD* gene and its *pCu$_A$C* neighbor (MettrDRAFT_3934-5) are 3-fold up-regulated by 5 h and 4-5-fold up-regulated by 24 h; the increased expression of this *pmoD* is statistically significant, and both these genes and the MettrDRAFT_0710 *pmoD* exhibit some correlation with the *pmo* operon and *csp1*. As previously observed, all components of the *pmo* operon do not necessarily exhibit a statistically significant copper response (even when co-regulation occurs)[6,7]; regulation of *pmo* and *amo* operons is complex and involves multiple overlapping transcription start sites and transcripts. In these experiments, no statistically significant copper-responsive changes in expression are observed for the in-operon MettrDRAFT_0381 *pmoD*, and greater correlation with the neighboring *copC*

(MettrDRAFT_0380) and *pmoB* (MettrDRAFT_0382) has been seen previously)[6,7]. Several *pmoD* genes exhibit no signs of any copper response: no statistically significant changes in expression or correlation with known copper-regulated genes are observed for three lone homologues (MettrDRAFT_0780, MettrDRAFT_1174, and MettrDRAFT_3978). Although differences in experimental design render the comparison imperfect, genes in this experiment that exhibit statistically significant copper-responsive expression changes or co-regulation with known copper-regulated genes (that is, MettrDRAFT_0039, MettrDRAFT_0710, and MettrDRAFT_3934) exhibit similar trends in expression changes between the 0 min (0 μM Cu) and 24 h (12.5 μM Cu) timepoints when compared to cells grown at 0 μM Cu and 10 μM Cu)[7].

For *Methylosinus trichosporium* OB3b, there is no clear correlation between copper-dependent gene regulation, sequence motif, and genomic neighborhood. For example, there are *pmoDs* that fall into the Cx_7Mx_nC category that are copper up-regulated and others that are mildly copper-repressed; similarly, there are both copper-repressed and mildly copper-up-regulated Cx_nC *pmoD*s (Supplementary Figure 8). Unlike their counterparts in *pmo*/*amo* operons, a *pmoD*/*copC* gene pair is copper-repressed in both *Methylosinus trichosporium* OB3b and *Methylococcus capsulatus* (Bath)[8], which is consistent with increasing evidence that at least some CopCs are related to copper import and not export (including the non-canonical C_{0-1} CopCs found in some *pmo*/*amo* operons, which bind a single Cu(II) ion)[9]. In contrast, a *pmoD*/*pCu$_A$C* pair in *Methylosinus trichosporium* OB3b and a *pmoD*/*MCO* pair in *Methylococcus capsulatus* (Bath) are copper-activated.

Genomic co-occurrence of operons with proposed roles in (methanotroph) copper homeostasis

As shown in Fig. 4a, the strongest reciprocal relationship is observed between pMMO/AMO and related enzymes and PmoD: 62.54 % of species that have CuMMO operons also have *pmoD* genes and 94.26 % of species with *pmoD* genes have CuMMO operons. Species whose genomes contain CuMMO-encoding operons but not *pmoD* genes are broadly members of three categories: ammonia-oxidizing archaea, proteobacterial species outside of canonical methanotroph genera that appear to have acquired pMMO homologues via horizontal gene transfer, and species that produce HMOs (hydrocarbon monooxygenases). By comparison to the correlation between CuMMOs and A/PmoD, genes encoding MopE and sMMO have limited distribution outside of species with CuMMO operons, while Mbn, Csp1/2, and Csp3 are all conversely found primarily in non-methanotrophs, exhibiting limited correlation with each other or with species that produce CuMMOs. Most overlap is due to the presence of genes encoding Csp1/2 and Csp3 homologues in the *Methylosinus* and *Methylocystis* genera, whose members all produce pMMO, mostly produce Mbns, and often produce sMMO.

Analysis of *csp1/csp2* and *csp3* genes requires an additional clarification. Membership in pre-existing uncharacterized protein families has not been discussed previously for the products of these genes[10]. However, Csp1 and Csp2 proteins appear to correlate quite well with the TAT-exported TIGR04401 family (and as recently noted, do not comprise two separate families[10]), while Csp3s may overlap completely with or comprise a subset of the DUF326 (PF03860) family. Although some *csp3* genes, such as the gene encoding *Methylosinus trichosporium* OB3b Csp3, are not annotated as members of the DUF326 family, analysis using tools such as InterProScan suggests that most or all of these proteins can nevertheless be classified as DUF326 proteins. Some more divergent members of the TIGR04401 family are also annotated as DUF326 proteins. Previous bioinformatics analyses relied on BLAST and T-Coffee multiple

sequence alignments (weighted towards methanotroph sequences). Nevertheless, as discussed in a recent review[10], other members of the DUF326 family (including Gram negative *Sphingobium* and *Pseudomonas* species and Gram positive *Bacillus* and *Streptomyces* species) are described as Csp3s, and a *Neisseria gonorrhoeae* TIGR04401 protein is described as having similar copper binding characteristics to *Methylosinus trichosporium* OB3b Csp1, lending support to the preliminary assignment of the Csp proteins to these two existing families.

Supplementary References

1. Blackburn, N. J. *et al.* X-ray absorption studies on the mixed-valence and fully reduced forms of the soluble Cu_A domains of cytochrome *c* oxidase. *J. Am. Chem. Soc.* **119**, 6135-6143 (1997).
2. Lieberman, R. L. *et al.* Characterization of the particulate methane monooxygenase metal centers in multiple redox states by X-ray absorption spectroscopy. *Inorg. Chem.* **45**, 8372-8381 (2006).
3. Riggs-Gelasco, P. J., Stemmler, T. L. & Penner-Hahn, J. E. XAFS of dinuclear metal sites in proteins and model compounds. *Coord. Chem. Rev.* **144**, 245-286 (1995).
4. Cotelesage, J. J., Pushie, M. J., Grochulski, P., Pickering, I. J. & George, G. N. Metalloprotein active site structure determination: synergy between X-ray absorption spectroscopy and X-ray crystallography. *J. Inorg. Biochem.* **115**, 127-137 (2012).
5. George, G. N. & Pickering, I. J. in *Brilliant light in life and materials sciences. NATO security through science series.* (eds Vasii Tsakanov & Helmut Wiedemann) 97-119 (Springer, 2007).
6. Kenney, G. E., Sadek, M. & Rosenzweig, A. C. Copper-responsive gene expression in the methanotroph *Methylosinus trichosporium* OB3b. *Metallomics* **8**, 931-940 (2016).
7. Gu, W. Y. & Semrau, J. D. Copper and cerium-regulated gene expression in *Methylosinus trichosporium* OB3b. *Appl. Microbiol. Biotechnol.* **101**, 8499-8516 (2017).
8. Larsen, O. & Karlsen, O. A. Transcriptomic profiling of *Methylococcus capsulatus* (Bath) during growth with two different methane monooxygenases. *Microbiologyopen* **5**, 254-267 (2016).
9. Lawton, T. J., Kenney, G. E., Hurley, J. D. & Rosenzweig, A. C. The CopC Family: structural and bioinformatic insights into a diverse group of periplasmic copper binding proteins. *Biochemistry* **55**, 2278-2290 (2016).
10. Dennison, C., David, S. & Lee, J. Bacterial copper storage proteins. *J. Biol. Chem.* **293**, 4616-4627 (2018).
11. Dassama, L. M., Kenney, G. E., Ro, S. Y., Zielazinski, E. L. & Rosenzweig, A. C. Methanobactin transport machinery. *Proc. Natl. Acad. Sci. USA* **113**, 13027-13032 (2016).
