# Supplementary material for: Characterization of a long overlooked copper protein from methane- and ammonia-oxidizing bacteria
Source: Nat Commun. 2018 Oct 15;9:4276. doi: 10.1038/s41467-018-06681-5 (PMC6189053; doi:10.1038/s41467-018-06681-5)
Supplement: Supplementary file 2 — Description of Additional Supplementary Files [file 41467_2018_6681_MOESM2_ESM.pdf]

Description of Additional Supplementary Files

File Name: Supplementary Data 1
Description: Primer efficiency calculations, NRQ calculations, and statistical analyses for qPCR experiments (.xls file)

File Name: Supplementary Data 2
Description: Trimmed list of amino acid sequences of PmoD homologues (.fa file)

File Name: Supplementary Data 3
Description: Trimmed dataset and metadata used to analyze and PmoD sequence similarity and genome neighborhood networks (.xls file)

File Name: Supplementary Data 4
Description: Hidden Markov Model for PmoD generated using HMMER 3.1 (.hmm file)

File Name: Supplementary Data 5
Description: PmoD sequence similarity network generated using the EFI-EST tool (.cys file)
